# A Genome-Wide Analysis Reveals No Nuclear Dobzhansky-Muller Pairs of Determinants of Speciation between *S. cerevisiae* and *S. paradoxus*, but Suggests More Complex Incompatibilities

**DOI:** 10.1371/journal.pgen.1001038

**Published:** 2010-07-29

**Authors:** Katy C. Kao, Katja Schwartz, Gavin Sherlock

**Affiliations:** Department of Genetics, Stanford University, Stanford, California, United States of America; Academia Sinica, Taiwan

## Abstract

The Dobzhansky-Muller (D-M) model of speciation by genic incompatibility is widely accepted as the primary cause of interspecific postzygotic isolation. Since the introduction of this model, there have been theoretical and experimental data supporting the existence of such incompatibilities. However, speciation genes have been largely elusive, with only a handful of candidate genes identified in a few organisms. The *Saccharomyces sensu stricto* yeasts, which have small genomes and can mate interspecifically to produce sterile hybrids, are thus an ideal model for studying postzygotic isolation. Among them, only a single D-M pair, comprising a mitochondrially targeted product of a nuclear gene and a mitochondrially encoded locus, has been found. Thus far, no D-M pair of nuclear genes has been identified between any *sensu stricto* yeasts. We report here the first detailed genome-wide analysis of rare meiotic products from an otherwise sterile hybrid and show that no classic D-M pairs of speciation genes exist between the nuclear genomes of the closely related yeasts *S. cerevisiae* and *S. paradoxus*. Instead, our analyses suggest that more complex interactions, likely involving multiple loci having weak effects, may be responsible for their post-zygotic separation. The lack of a nuclear encoded classic D-M pair between these two yeasts, yet the existence of multiple loci that may each exert a small effect through complex interactions suggests that initial speciation events might not always be mediated by D-M pairs. An alternative explanation may be that the accumulation of polymorphisms leads to gamete inviability due to the activities of anti-recombination mechanisms and/or incompatibilities between the species' transcriptional and metabolic networks, with no single pair at least initially being responsible for the incompatibility. After such a speciation event, it is possible that one or more D-M pairs might subsequently arise following isolation.

## Author Summary

Species are defined such that organisms of the same species can produce fertile offspring, whereas organisms of different species are either unable to mate, or when they do, they produce inviable or sterile progeny. A well-known pair of species that can mate yet produce sterile offspring is the horse and donkey, which produce an infertile hybrid, the mule. A long-standing idea for the species barrier is that when certain pairs of genes from the two different species are combined, the genes can no longer function properly, thus causing death or sterility. Identification of these incompatible genes may allow us to determine how organisms form distinct species, and understand the process of speciation itself. We used two closely related yeasts to look for these incompatible genes by isolating rare viable hybrid offspring, and looking for excluded gene combinations. We did not find any pairs of incompatible genes, but instead found that there appear to be more than two genes involved in such incompatibilities. We speculate that the accumulation of large numbers of sequence differences in their DNA may cause defects in how genes are controlled in hybrids, causing these two yeasts to be independent species.

## Introduction

Dobzhansky and Muller independently proposed the genic incompatibility model as the genetic basis for the barrier to gene flow in postzygotic speciation [1], [2], whereby epistatic interactions at two or more loci between two species can cause sterility or inviability in a hybrid organism. Their model became known as the Dobzhansky-Muller (D-M) model of speciation, with the simplest form of the model involving interaction of a pair of genes, referred to as a D-M pair. This genic incompatibility model of postzygotic speciation has been widely accepted and has been supported both theoretically and experimentally by a large body of literature [3], [4], [5], [6], [7], [8], [9]. However, the identities of these speciation genes have largely remained elusive. Only a few genes involved in reproductive isolation have been identified, mostly in *Drosophila* [5], [6], [7], [10], [11], [12]. Most speciation genes identified have been either located on the X chromosome or are incompatible with loci on the X chromosome, consistent with Haldane's rule [13]. For example, the Odysseus gene (*OdsH*), on the X chromosome in *Drosophila*, causes hybrid male sterility between *D. simulans* and *D. mauritiana* [5]. The *D. mauritiana* OdsH protein was recently shown to localize to and interact with the Y chromosome of *D. simulans*, possibly causing decondensation of the heterochromatin, resulting in hybrid sterility [14]. The *D. simulans* nucleoporin-96, *NUP96*, is incompatible with an unknown allele on the X chromosome of *D. melanogaster* [7]. The identity of the first pair of interacting D-M genes were recently reported in *Drosophila*, where the *Lethal hybrid rescue* (*LHR*) gene in *D. simulans* is incompatible with the *Hybrid male rescue* (*HMR*) gene from *D. melanogaster* [10]. Recently, a speciation gene in mice was identified to be *Prdm9*, which encodes a meiotic histone H3 lysine 4 trimethyltransferase [15].

The members of the *Saccharomyces sensu stricto* group of yeasts provide an ideal model system for investigating the molecular mechanisms of speciation. There are currently six known members of the *Saccharomyces sensu stricto* group, with *S. paradoxus* being the closest relative of *S. cerevisiae*, with an overall DNA sequence identity of approximately 85%, and *S. bayanus* being the farthest relative with an overall sequence identity of approximately 62%. The members of the *Saccharomyces sensu stricto* group of yeast can mate readily, where two haploid strains from different species and of the opposite mating type can form a viable heterozygous hybrid diploid (F_1_ hybrid). Such F_1_ hybrids can undergo meiosis (sporulation) to produce spores (haploid gametes), but the spore viability is less than 1% [16], [17]. Thus, the *Saccharomyces sensu stricto* yeasts are considered to be postzygotically isolated. In addition to postzygotic isolation, studies have shown potential mating preferences between *S. cerevisiae* and *S. paradoxus* [18], suggesting that prezygotic isolation also plays a role in the reproductive isolation of these yeasts. It has been shown that hybrids made between members of the *Saccharomyces sensu stricto* group can produce rare viable progeny, and that these progeny themselves are postzygotically separated from one another and their parents [19], and thus, by the classic definition, are distinct species. Studies of the *Saccharomyces sensu stricto* yeasts have shown that genome rearrangements [20], [21] and the mismatch repair system [17], [22] contribute to the mechanisms of postzygotic isolation between different species in this group. However, prior work has suggested that dominant genic incompatibilities do not exist between *S. cerevisiae* and *S. paradoxus* [16], and a recent effort to identify recessive genic incompatibilities between these two species, by replacing individual chromosomes from *S. cerevisiae* with the *S. paradoxus* versions, was unable to identify any such incompatibilities [23]. However, in that study only 9 of the possible 16 chromosomal replacements could be made and the resulting strains did not undergo meiosis and germination, thus not making it possible to conclusively determine whether any recessive genic incompatibilities exist as a reproductive barrier in hybrids between the two species. Most recently, a D-M pair of interacting genes was identified in the *Saccharomyces sensu stricto* yeasts. Hybrids were generated by replacing chromosomes in *S. cerevisiae* with the corresponding ones from *S. bayanus*, and the homozygous diploid hybrid was created via self-fertilization, which was then tested for sterility [12]. The identified incompatibility involved a nuclear encoded gene from *S. bayanus*, *AEP2*, whose product is mitochondrially targeted, and a mitochondrial gene encoding an ATP synthase subunit in *S. cerevisiae*, *OLI1* [12], whose 5′ UTR is bound and regulated by Aep2. Cells containing the incompatible pair are unable to respire and thus unable to sporulate. Unlike most of the other speciation genes identified so far, the *AEP2* gene does not appear to be under positive selection, suggesting that positive selection may not be a criterion for genic incompatibility. However, due to several reciprocal translocations between *S. cerevisiae* and *S. bayanus*, Lee *et al* [12] were not able to examine the effects of all the individual chromosomes, since these translocation-containing chromosomes needed to be replaced together, which was technically infeasible. In addition, the presence of any genic incompatibilities that involve multiple loci (residing on different chromosomes) would likely not have been detected via the replacement of individual chromosomes.

Thus far, no comprehensive, genome-wide effort has been made to determine whether D-M genic incompatibilities (at least between nuclear genomes) play a role in the postzygotic isolation between members of the *Saccharomyces sensu stricto* group. We have exploited the complete genome sequences of *S. cerevisiae* and *S. paradoxus* [24], [25] to take a novel approach to identifying such loci. We have used these genome sequences to design dual species microarrays for Comparative Genome Hybridization (array-CGH) to assay the genomes of rare viable F_1_ spores at high resolution to locate potential speciation loci. Our hypothesis is that there exist genetic determinants in *S. cerevisiae* and *S. paradoxus* that are incompatible, resulting in failure of spores to germinate or form colonies. This study differs from the study by Lee *et al*, which in essence looked at the fertility of the F_2_ gametes. To determine whether D-M genic incompatibilities exist in their nuclear genomes, we assayed the genome content of more than one hundred rare viable spores produced from F_1_ hybrids between *S. paradoxus* and *S. cerevisiae*. If Dobzhansky-Muller type genic incompatibilities exist between these species, we would expect to see patterns in the genome contents of the viable F_1_ spores, where combinations of incompatible loci will be excluded or at least underrepresented in the viable F_1_ spores. Our results show that there are no simple classic D-M pairs of interacting genes between the nuclear genomes of the two species. However, we do identify some underrepresented combinations of loci, and these combinations typically involve more than two loci, suggesting more complex D-M interactions. We also find chromosome 4 to be preferentially inherited from *S. cerevisiae*, indicative of the presence of a potential incompatible locus on this chromosome. Our results suggest that genic incompatibilities within the nuclear genomes between members of the *Saccharomyces sensu stricto* yeasts involve multiple incompatible loci, with weak individual effects.

## Results

To identify candidate speciation genes in the *Saccharomyces sensu stricto* yeast, we used *S. cerevisiae* and *S. paradoxus* as the parental species, since their genomes are essentially collinear with no gross chromosomal rearrangements between them [24], eliminating chromosomal rearrangements as a major contributor of postzygotic isolation in our study. The mismatch repair system has been shown to play a role in the reproductive isolation between these two species [22], [26]; however, it is not the sole contributor to hybrid sterility in these organisms, as the viability of hybrid spores in the mismatch repair deficient strains is still only 10% [17] (it is not clear how much of the remaining sterility may be explained by mismatch repair independent anti-recombination mechanisms). We thus derived rare F_1_ spores from both mismatch repair proficient and deficient (due to *MSH2* deletion) F_1_ hybrids of *S. cerevisiae* and *S. paradoxus*, and their genome contents were then determined using array CGH.

### Dual-species array-CGH analysis of hybrids

**Figure 1:**
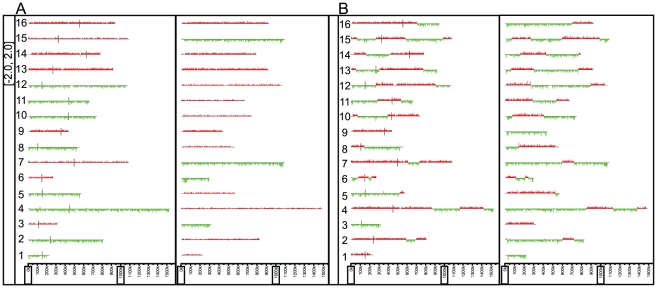
Example karyoscopes of viable F_1_ spores. A) Wild-type F_1_ derived spore and B) mismatch repair deficient F_1_ derived spore.

Dual species DNA microarrays were designed for *S. cerevisiae* and *S. paradoxus* for the determination of the genomic contents of the viable F_1_ spores. The microarray contains 7,134 *S. cerevisiae* probes and 7,047 *S. paradoxus* probes, at a resolution of approximately 2 kb across both genomes. The array also contains probes that were designed based on the sequence of the *S. cerevisiae* mitochondrion. The 60-mer oligonucleotide probes were chosen such that they were best able to distinguish between the two parental genomes (see Materials and Methods for details of probe design and microarray validation). Using these arrays, we interrogated the genomes of 58 spores derived from two independent mismatch repair proficient F_1_ hybrids, and 48 spores derived from two independent mismatch repair deficient F_1_ hybrids. Using the software Caryoscope [27], we visualized which portions of the genome were inherited from which parent (either *S. cerevisiae* or *S. paradoxus*) (see Figure 1A and B for examples).

### Extensive aneuploidy and reduced recombination observed in the spores derived from mismatch repair proficient F_1_ hybrids

The F_1_ spores derived from mismatch proficient parents showed extensive aneuploidy (defined here as the presence of a particular chromosome from both parental species), with the majority of the genomes assayed containing at least one, and up to five, aneuploid chromosomes. In addition, the rate of recombination was also reduced in these F_1_ spores, with an average of only 2.7 crossovers observed per viable spore, confirming previous reports that chromosome nondisjunction, due to the mismatch repair system preventing homeologous recombination, may be involved in F_1_ sterility in the *Saccharomyces* yeasts [17], [26]. Mismatch repair mutants have been shown to increase recombination between homeologous chromosomes [17], [22]. The spores derived from mismatch repair deficient F_1_s (generated by deleting *MSH2*) showed a dramatic decrease, of approximately 10 fold compared to the wild-type, in the number of aneuploid chromosomes per strain. The number of recombinations also increased by more than 6 fold, to an average of 17.8 recombinations, per strain, which is approximately one per chromosome (see Table 1), though lower than would normally be seen in a non-hybrid strain (17.8 recombination events compared to 39 per spore in intraspecific meiotic products, as reported in Mancera et al [28]). The assayed F_1_ spores between *S. cerevisiae* and *S. paradoxus* showed no overall bias in inheritance of the genome from one parent over the other.

**Table 1:** The number of aneuploidy and recombination events observed in viable F_1_ spores.

Chromosome		1	2	3	4	5	6	7	8	9	10	11	12	13	14	15	16	Average*
Mismatch proficient F_1_ spores																		
Recombination																		
	Sc	4	11	1	23	12	5	12	5	4	10	8	14	15	9	13	10	2.66
	Sp	4	11	1	22	12	5	12	5	6	10	7	13	14	10	12	9	
Aneuploidy																		
	Sc	10	9	9	0	12	2	4	8	16	8	14	9	7	10	5	10	2.29
	Sp	9	9	9	1	12	2	4	8	14	8	14	9	8	10	5	11	
Mismatch repair mutant F_1_ spores																		
Recombination																		
	Sc	23	63	27	126	46	20	74	42	28	53	37	75	68	47	71	60	17.83
	Sp	19	62	27	124	45	20	74	42	27	53	37	72	71	48	70	61	
Aneuploidy																		
	Sc	4	2	0	0	0	0	0	2	2	0	1	1	1	0	0	1	0.29
	Sp	4	2	0	0	0	0	0	2	2	0	1	2	0	0	0	1	

Sc: *S. cerevisiae*.

Sp: *S. paradoxus*.

*The average is calculated based on the average recombination events and aneuploidies observed on both *S. cerevisiae* and *S. paradoxus* portion of the genomes.

**Figure 2:**
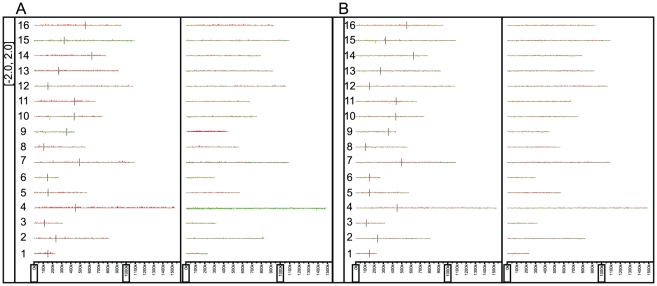
The karyoscopes of roughly 1,000 pooled F_1_ spores. A) Wild-type derived F_1_ spores and B) mismatch repair mutant derived F_1_ spores. The *S. cerevisiae* genome is on the left, with the *S. paradoxus* genome on the right.

From the 58 F_1_ spores derived from the wild-type F_1_ hybrid, we observed no crossovers in chromosome 4 in 39 wild-type spores, 30 of which had inherited the *S. cerevisiae* chromosome 4, while only 9 inherited the *S. paradoxus* chromosome 4. Using a binomial distribution, the *S. paradoxus* chromosome 4 appears to be underrepresented in the spores from the wild-type F_1_ hybrid, with a p-value of 0.0004. This was confirmed by array-CGH of pooled genomic DNA from roughly 1000 viable spores from a mismatch proficient F_1_ hybrid (Figure 2A), and has been replicated from independent F_1_ hybrids. Since the mismatch repair deficient hybrids have increased meiotic recombination, we performed a similar pooling experiment with the spores from the mismatch repair deficient F_1_ hybrids, but were unable to identify any specific region on chromosome 4 to be biased in its inheritance from one species (Figure 2B).

For each pair of homeologous chromosomes, we compared the sequence similarities, GC contents, frequencies of observed recombination in the viable F_1_ spores, and frequency of meiotic recombination in *S. cerevisiae* [28] (Figure S1). It has been observed that the recombination frequency in yeast tends to be higher in regions with higher GC content [29]. We calculated the GC content across each *S. paradoxus* and *S. cerevisiae* chromosome to see if there were dramatic differences between the local GC content between the two species, but found them to be highly similar (see Figure S1). Comparisons between regions with high local GC content and the local sequence similarity across each chromosome between the two species revealed no bias in low sequence similarity and high GC content (See Table S1). Lower sequence similarities between two homeologous chromosomes may result in lower frequency of recombination. However, as shown in Table S1, we found no correlations between overall sequence similarities and frequency of observed recombination in the viable F_1_ spores.

### Linkage analysis to identify potential loci involved in reproductive isolation

**Figure 3:**
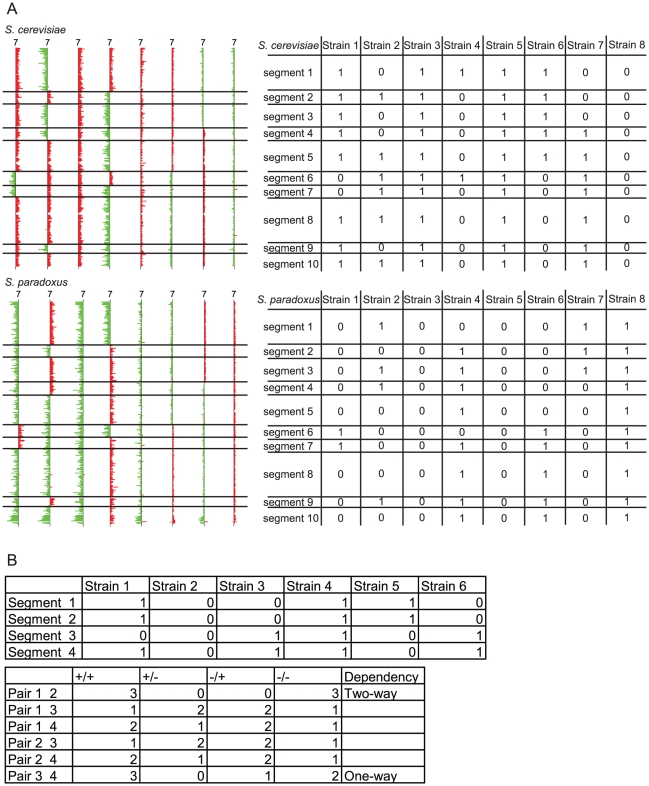
Example of the segmentation analysis process. A) Segmentation analysis of chromosome 7 for eight F_1_ spores. Chromosome 7 was divided into segments based on the recombination breakpoints shown on the left. The analysis was performed separately for *S. cerevisiae* and *S. paradoxus*. Each particular segment of the genome is translated into tables on the right, where 1 indicates the presence of, 0 indicates the absence of, and 2 indicates the presence of aneuploidy in the particular segment; B) Example of break down of categories for pairwise analysis for segment 4 and segment 7 for the *S. cerevisiae* portion of the genome.

The simplest form of the D-M model involves only two interacting loci; thus, to determine whether simple D-M pairs exist between *S. cerevisiae* and *S. paradoxus*, we performed pair-wise linkage analysis separately for each genome to determine if any two loci derived from one of the parent's genomes showed a dependency. Such a dependence would likely manifest as an altered pattern of segregation of the two loci from the same genome with respect to one another compared to what would be expected by chance, as determined using a Chi-square test. Our reasoning is that if there is a D-M pair, we are likely to observe these two loci being co-inherited from the same parental genome in rare viable F_1_ spores. Because we observed reduced meiotic recombination in the viable F_1_ spores, we expected that chromosomal segments on smaller chromosomes would be co-inherited anyway, while the segments within larger chromosomes would segregate randomly, depending on how far apart they were. By looking at the relationship between the distance between intrachromosomal segments and the Chi-square statistic between them, we found this to be mostly true (See Figure S2). For all 16 chromosomes, we found an inverse relationship between the distance between segments, and the Chi-square statistics between the pairs of segments within each chromosome. The minimum distance between loci within the same chromosome for which there is no significant linkage (significance as determined by an arbitrarily chosen FDR of 0.01) was approximately 180 kb. Thus, if there are potential dependencies between linked loci within approximately 180 kb of the same chromosome, we will not be able to identify them using our analysis. For the remainder of the analysis, we only performed linkage analysis between segments from different chromosomes. To perform this analysis, we first identified all the locations on each chromosome for both parental genomes where a meiotic recombination event had occurred in the production of any of the viable F_1_ spores assayed. We then segmented each chromosome in both genomes (*S. cerevisiae* and *S. paradoxus*) for each of the 106 F_1_ spores, at the observed recombination locations (irrespective of whether a given F_1_ spore had a recombination event at that location). For example, there were 19 observed recombination locations on the *S. cerevisiae* chromosome 1 across all F_1_ spores, resulting in 20 segments for the *S. cerevisiae* chromosome 1. After segmentation, each segment for each F_1_ spore was scored for its presence or absence in that strain for a particular parental species (*e.g. S. cerevisiae*), where the segment was given a score of 1 if present from *S. cerevisiae*, and 0 if present from *S. paradoxus*. If a segment is aneuploid (having inherited both *S. cerevisiae* and *S. paradoxus* sequences), then it was given a score of 2. The data for the chromosomes of each parental species were analyzed separately. An example is illustrated in Figure 3A. There were a total of 834 and 830 segments for *S. cerevisiae* and *S. paradoxus* genomes, respectively.

We analyzed these data, with each segment scored for its presence, absence, or aneuploidy for a particular parental species (as defined above), to determine the pattern of segregation for all pairwise combinations of segments within a parental genome (excluding segments on the same chromosome). There are a total of nine possible categories for each pair of segments. We initially excluded the categories that involved aneuploid segments, and thus each pair of segments from a given F_1_ spore was classified into one of four categories, with respect to the segments having been inherited from one or both of the parents (as shown in Figure 3B), for example: i) both segments present from *S. cerevisiae*, ii) one present from *S. cerevisiae* and one present from *S. paradoxus*, iii) vice versa, and iv) both from *S. paradoxus*. The total numbers of interchromosomal pair-wise comparisons were 322,258 and 318,545 for *S. cerevisiae* and *S. paradoxus* genomes, respectively. If there was no dependence between the loci in such a pair, random segregation of two loci would predict that the values for each of these categories would not be significantly different from the expected values. However, if the two loci contain genes that participate in a D-M pair between the two species, we would expect the distribution in the four categories to be skewed. For example, if there exist two loci, *A* and *B* in the *S. cerevisiae* genome, with alleles *a* and *b* from *S. paradoxus*, we would expect that in the case of a two-way dependency, only the parental genotypes, *AB* and *ab*, would be viable, with *Ab* and *aB* being inviable. In the case of a one-way dependency, we might observe that *A* is compatible with both *B* and *b*, but *a* is only compatible with *b*, where the only incompatible genotype is *aB*. In the two-way dependency case, where the two loci always need to be from the same species, the pairwise analysis for these two loci should reveal no entries in both categories ii and iii, since the presence of one locus from a given parent requires the presence of the other derived from the same parent to function. The one-way dependency scenario is what was found in most prior studies on reproductive isolation in *Drosophila* [30], [31] and yeast [12]. In the one-way dependency scenario, for the two loci, either category ii or iii would contain no entries. To determine significance, the chi-square test was used for the linkage analysis. To remove the bias in the frequency of inheritance of each segment, we normalized the expectation for the chi-square test. An expectation was calculated based on the number of F_1_ spores having inherited each segment. For example, if segment A and segment B were inherited from *S. cerevisiae* in 20 out of 106 and 50 out of 106 total F_1_ spores, respectively, then the probability of an F_1_ spore having inherited both A and B from *S. cerevisiae* would be (20/106)*(50/106) and the expectation for the number of F_1_ spores having inherited both segments would be (20/106)*(50/106)*106. For statistical significance, a false discovery rate (FDR) for each chi-square statistic was determined by permutation, randomizing each segment between the 106 strains and calculating the pair-wise chi-square statistics for each pair of randomized segments. The false discovery rate was then estimated by dividing the average number of pairs with a chi-square statistic greater than x in the randomized samples over 1000 iterations by the numbers observed in the data set (Tables S2 and S3 contain all the data).

### No simple D-M pairs

From this analysis, we found no pair of segments that have either a one-way or a two-way simple D-M dependency. That is, there were no pairs of segments from different chromosomes for which any of the patterns of inheritance were excluded, clearly demonstrating the absence of any simple D-M pairs of incompatible genes on different chromosomes within the nuclear genomes between *S. cerevisiae* and *S. paradoxus*. However, our linkage analysis revealed several pairs of segments that may be involved in more complex D-M genic incompatibilities involving more than two loci, as these segments show distributions that are statistically significantly different than what would be expected by chance, using an FDR of 0.01. Statistically significant pairs of regions of the *S. cerevisiae* and *S. paradoxus* genomes are shown in Tables S4 and S5. In addition, we also analyzed the entire dataset (all nine categories, including the aneuploid segments). However, due to the large number of categories, we did not have sufficient power to identify any significant pairs of segments using an FDR of 0.01.

### More complex interactions

While our data revealed no simple D-M pairs of interacting genes, the data do suggest more complex genic incompatibilities, involving more than 2 loci. The results of *S. cerevisae* and *S. paradoxus* genomes were reciprocal to one another, as expected. If these incompatibilities include two-way dependencies, then we would expect categories ii and iii to have similar behaviors (similar deviations from the expected). Several pairs of chromosomes show potential two-way dependency. Chromosome pairs 1-10, 2-14, 3-13, 4-8, and 7-16 are more likely to be co-inherited from the same parent (FDR <0.01), suggesting potential dependencies involving loci residing on these chromosomes. Interestingly, chromosomes 2 and 9, chromosomes 4 and 13, and chromosomes 11 and 14 are less likely to be both inherited from the same parent (FDR <0.01). Approximately 12% of the nuclear genome is involved in potential interactions.

To estimate whether there exist any 3 interacting loci that may be involved in F_1_ hybrid spore inviability, we conducted linkage analysis for all possible combinations of 3 loci (A, B, and C). There were a total of 8 categories: 1) all 3 loci present from *S. cerevisiae*, 2) A, B from *S. cerevisiae*, but C from *S. paradoxus*, 3) A and C from *S. cerevisiae*, but B from *S. paradoxus*, 4) A from *S. cerevisiae*, B and C from *S. paradoxus*, 5) B from *S. cerevisiae*, A and C from *S. paradoxus*, 6) A from *S. paradoxus*, B and C from *S. cerevisiae*, 7) C from *S. cerevisiae*, A and B from *S. paradoxus*, and 8) all 3 loci from *S. paradoxus*. Linkage analysis between all possible combinations of 3 loci identified 138,322 groups with a zero entry in any one of the categories (not taking into account aneuploidies) (compared to an average of 28,081 from permutations analysis; data not shown), indicative of potential dependencies between the loci. However, due to an insufficient sample size, we cannot confidently determine whether these categories are truly excluded from the viable F_1_ spores, or whether what we have observed has simply arisen by chance.

### Growth on non-fermentable carbon source

We tested the F_1_ spores for the ability to grow on glycerol as their sole carbon source. Approximately 85% of the F_1_ spores were able to grow on the non-fermentable carbon source. Thus, at least 15% of the spores will form sterile F_2_ zygotes. This suggests the presence of incompatibilities between the nuclear genome and the mitochondrial DNA between the two species (or the absence of mitochondrion), as these particular combinations of the *S. cerevisiae* and *S. paradoxus* genomes did not allow the resulting F_1_ spore to grow on non-fermentable carbon source. However, the small number of spores with this phenotype precluded the identification of any loci that might be responsible for this incompatibility.

## Discussion

### A genome-wide assessment of incompatibilities

Our data represent the first comprehensive genome-wide effort to determine genic incompatibility, which is responsible for failure of F_1_ spores to germinate and form colonies, between members of the *Saccharomyces sensu stricto* yeasts. We found no simple Dobzhansky-Muller pair of speciation genes within the nuclear genomes of *S. cerevisiae* and *S. paradoxus*. Prior reports have suggested that neither dominant nor recessive genic incompatibilities exist between members of the *Saccharomyces sensu stricto* group of yeasts [16], [23], and our data further confirm this. A recent survey of sequence variation in subpopulations of *S. paradoxus* and their gamete viabilities in crosses between different isolates revealed a direct correlation between sequence divergence and spore viabilities [32], further supporting the notion that sequence divergence plays a major role in the reproductive isolation between the *Saccharomyces sensu stricto* yeasts. Thus, the current predominant theory regarding postzygotic speciation in this group of yeasts is the failure of proper segregation due to the mismatch repair system [17], [23]. However, even in mismatch repair deficient *S. cerevisiae* and *S. paradoxus* F_1_ hybrid, the spore viability was still approximately 10% [17], leaving a large percentage of inviability unexplained by mismatch repair system alone. Reduced frequency of recombination caused by mismatch repair system independent anti-recombination mechanisms [33] may also contribute to the reduced spore viability of F_1_ hybrids.

### Multiple interacting loci identified

The first interacting pair of speciation genes was recently identified between the mitochondrion of *S. cerevisiae* and a nuclear gene in *S. bayanus* [12]; incompatibilities between the nuclear genome and the mitochondria between other members of the *Saccharomyces sensu stricto* group were apparently also observed, but not detailed. Our data support the presence of speciation genes involving the nuclear genomes of *S. cerevisiae* and *S. paradoxus*, but these are complex interactions involving multiple loci. While it is possible that the presence of speciation genes are masked by compensatory mutations in the viable spores, the mutation rate of approximately 45×10^−8^ [34] suggests that it would be unlikely in our study. If no complex genic incompatibilities (or if the effects of the incompatibilities were insignificant) exist between these two species, then we would have expected no pairs of statistically significant loci from our linkage analysis of the 106 viable F_1_ spores. Instead, we identified several loci having segregation distributions that significantly differ from expectation, indicative of more complex interactions likely involving multiple loci. Assuming that these complex interactions involve groups of 3 speciation genes, then we would expect there to exist 7–8 groups of 3 interacting loci for a reduction of 88%–100% in hybrid spore viability. Among the 106 F_1_ hybrid spores analyzed, we identified 138,322 groups of 3 loci that showed potential dependencies (compared to an average of 28,081 from permutation analysis). However, due to insufficient sample size, we cannot confidently conclude that these dependencies exist. It is however clear that the presence of multiple potential interacting pairs of loci identified in the viable spores of F_1_ hybrids is indicative of the involvement of multiple loci with weak effects, rather than the involvement of few loci with strong effects, contributing to genic incompatibilities between these two species. Similar “multilocus weak allele interactions” were also observed in studies of reproductive isolation in *Drosophila* [35].

### Chromosome 4 from *S. cerevisiae* is preferentially inherited in viable F_1_ spores

Interestingly, we found the *S. cerevisiae* copy of chromosome 4 to be preferentially inherited by the viable F_1_ spores, based on both statistical analysis of the viable spores derived from mismatch repair proficient F_1_ hybrids and verification by pooling approximately 1000 viable spores of F_1_ wild-type hybrids in two independent crosses (Figure 2A). Unfortunately, attempts to narrow down the region on chromosome 4 that is preferentially inherited from *S. cerevisiae* by pooling spores from the mismatch repair deficient F_1_ parent failed to reveal the identity of the significant locus on this chromosome (Figure 2B). This discrepancy between the mismatch repair proficient and deficient F_1_ hybrid spores may a result of the significantly increased rate of recombination (approximately 6 fold) in the mismatch repair deficient F_1_ hybrids. For example, if such an incompatibility involves chromosome 4 and two loci, A and B, on another chromosome (we would not be able to detect these due to the tight physical linkage of intrachromosomal segments as described in the Results section), A and B would typically be co-inherited in the mismatch repair proficient F_1_, due to a lack of recombination. However, the increased recombination in the mismatch repair deficient F_1_ hybrid will dramatically decrease the probability of both A and B being inherited from *S. cerevisiae*, and result in the lack of preferential inheritance of chromosome 4 from *S. cerevisiae* in the pooled mismatch repair deficient F_1_ spores. In addition, even though extensive aneuploidy was observed in the spores from wild-type F_1_ hybrids, with most chromosomes showing multiple aneuploidy events, the number of aneuploidies observed for chromosome 4 was significantly lower than would be expected by chance (Bonferroni corrected p-value of 0.02 using a binomial distribution), with only a single observed event (See Table 1). It is the only chromosome to exhibit a statistically significantly lower rate of aneuploidy (using a corrected p-value cut-off of 0.05). Difficulties in isolating *S. cerevisiae* strains aneuploid for certain chromosomes (most notably in chromosomes 4 and 6) have been observed previously [36], [37], [38]. Lethality due to an extra copy of chromosome 6 has been partly attributed to imbalance in the copy number of the beta-tubulin gene, *TUB2*, which resides on chromosome 6, to that of the alpha-tubulin genes, *TUB1* and *TUB3*, which reside on chromosome 13 [36], [39]. Among the 58 F_1_ spores generated from mismatch repair proficient hybrids, we only observed 2 aneuploidies in chromosome 6; however, this was not statistically significant after Bonferroni correction. Aneuploidy in chromosome 4 has been shown to cause longer delay in entry into cell cycle [37] and has been attributed to the extra burden of protein synthesis due to an extra copy of the largest chromosome. Thus, it is possible that hybrids containing extra copies of chromosome 4 were selected against due to their slower growth rates.

### Postzygotic isolation between *S. cerevisiae* and *S. paradoxus* possibly caused by the effects of multiple interactions combined with transcriptional regulatory network perturbations

The lack of any simple pair-wise genic incompatibilities between the nuclear genomes, and the identification of multiple significant pairs of regions may be indicative that postzygotic isolation is due to the combined effects of multiple interactions, each with small effects. Examining known interactions between genes within the significant pairs of loci, we found several pairs of segments containing multiple pairs of genes with known interactions (see Table S6). Thus, it is possible that the sum of all incompatible pairs (no matter how small the effect) inherited by the F_1_ spores plays a bigger role in the reproductive isolation between these two species than simple D-M genic incompatibilities. However, it is noteworthy that a recent study on incipient speciation in *Neurospora* generated from divergently evolved populations identified a two-loci asymmetric interaction that resulted in a large decrease in meiotic efficiency [40].

Gene expression regulation has been implicated as a mechanism for reproductive isolation in *Drosophila* interspecific hybrids [41], [42], [43]. Our recent work demonstrated that even a single nucleotide change in the yeast genome can result in large changes in the global transcriptional profiles [44]. With the genome sequences between *S. cerevisiae* and *S. paradoxus* being diverged by approximately 15% in the intergenic regions and approximately 10% in the coding regions, it is likely that there has been significant rewiring in the transcriptional regulatory network, including both *cis* and *trans* regulatory changes, that may contribute significantly to reproductive isolation in the *Saccharomyces sensu stricto* yeasts. Indeed, recent work has demonstrated that in F_1_ hybrids between *S. paradoxus* and *S. cerevisiae* that there are significant *cis* and *trans* regulatory differences [45]. Thus, the potential interacting loci identified from our analyses may not necessarily be involved in functional or physical interactions, but may be involved in the proper timing and regulation of gene expression. Even though we were not able to identify the exact genes involved, due to the loci containing multiple genes, these significant pairs of loci identified in our studies will be helpful in narrowing down potential candidates with additional research.

Recent work showed that when clones derived from haploid populations of *S. cerevisiae* that have evolved for 500 generations in either high saline or low glucose conditions were crossed, the resulting diploid had a reduced sporulation efficiency [46]. This inferred “incipient speciation” was not seen when crossing clones independently evolved under the same conditions. Our earlier work has shown that adaptive clones derived from haploid *S. cerevisiae* evolved under glucose-limited conditions for approximately 450 generations have only a handful (certainly less than 10) of mutations ([44] and G. Sherlock and D. Kvitek, unpublished results). It is likely that comparable small numbers of mutations exist in the clones derived by Dettman *et al* [46] in their laboratory evolved populations; thus, the reduced meiotic efficiencies observed in their work are unlikely to have arisen due to classic D-M interacting proteins, but may be due to a few mutations causing large and incompatible changes in the transcriptional networks. Therefore, in addition to anti-recombination mechanisms, the 15% sequence divergence between *S. cerevisiae* and *S. paradoxus* likely results in two possible mechanisms of incompatibilities: 1) combinations of multiple potential genic incompatibilities with small effects (as no simple D-M pair was identified from our analysis) and 2) transcriptional regulatory network effects due to misregulation in the level and timing of expressions of genes in hybrid F_1_ spores, whose network will contain a mix of parts from both parents. It is unclear whether observed classic D-M pairs are frequently the cause of speciation, or whether they arise after the fact, due to these other factors that successively reduce hybrid fertility.

## Materials and Methods

### Yeast strains

The yeast strains used are listed in Table 2. All *S. cerevisiae* strains are derivatives of S288c. All *S. paradoxus* strains used are derivatives of the sequenced type strain CBS432 (NRRL Y-17217).

**Table 2:** List of strains.

Strain	Species	Genotype	Comment	Source
GSY82	*S. paradoxus*	Mat? *ura3-1*/*ura3-1*	CBS432	Ed Louis
GSY83	*S. cerevisiae*	Mat? *ade2-1 lys2* ho	S288c	Ed Louis
GSY88	*S. paradoxus/S. cerevisiae*	Mat**a**/**α** *ura3-1*/*URA3 ADE2*/*ade2-1 LYS2*/*lys2* HO/ho	GSY82× GSY83	Ed Louis
GSY145	*S. cerevisiae*	Mat**α** ho		
GSY147	*S. cerevisiae*	Mat**a** ho		
cc154	*S. paradoxus*	Mat**a** ho::KAN		Ed Louis
cc154× GSY145	*S. paradoxus/S. cerevisiae*	Mat**a**/**α** ho::KAN/ho	F_1_ hybrid	This work
GSY896	*S. cerevisiae*	Mat**a** *ura3-52 leu2Δ ade2*		
GSY145×GSY896	*S. cerevisiae*	Mat**a**/**α** ho/ho *URA3*/*ura3-52 LEU2*/*leu2Δ ADE2*/*ade2*		This work
Sc_msh2_ko_1 12B	*S. cerevisiae*	Mat? *msh2*::KAN ho leu2Δ		This work
Sc_msh2_ko_6 15B	*S. cerevisiae*	Mat? *msh2*::KAN ho leu2Δ		This work
Sp_msh2_ko_1 7A	*S. paradoxus*	Mat? *msh2*::KAN *ura3-1*/*ura3-1* HO/HO		This work
Sp_msh2_ko_2 7B	*S. paradoxus*	Mat? *msh2*::KAN *ura3-1*/*ura3-1* HO/HO		This work
Sc_msh2_ko_1 12B × sp_msh2_ko_1 7A	*S. cerevisiae/S. paradoxus*	Mat**a**/**α** *msh2*::KAN/*msh2*::KAN *leu2Δ*/*LEU2 URA3*/*ura3-1* ho/HO	Parent of the P5 F_1_ spores	This work
Sc_msh2_ko_6 15B × Sp_msh2_ko_2 7B	*S. cerevisiae/S. paradoxus*	Mat**a**/**α ** *msh2*::KAN/*msh2*::KAN *leu2Δ*/*LEU2 URA3*/*ura3-1*, ho/HO	Parent of the P6 F_1_ spores	This work

### Generation of *MSH2* mutants

To generate the *msh2* mutants, the 5′ and 3′ regions of the *msh2* genes in *S. cerevisiae* and *S. paradoxus* were amplified by PCR. These PCR products were fused to KanMX6 from pFA6-KanMX6 [47] via crossover PCR. Diploid heterozygous mutants in *msh2* were generated by transforming the resulting fragments for *S. cerevisiae* and *S. paradoxus* into the diploid *S. cerevisiae* strain GSY145×GSY896 or the diploid *S. paradoxus* strain GSY82 (See Table 2 for genotype), respectively, via a lithium acetate method [48] and plated on YPD plates containing 200 µg/ml G418. Two independent successful transformants (*msh2*::KAN/*MSH2*) were chosen for each species and verified via colony PCR using the species-specific verification primers listed in Table 3. These chosen transformants were sporulated in sporulation medium (1% potassium acetate and 0.02% raffinose) for 3 days and resulting spore products were screened for G418 resistance. Since the *S. paradoxus* diploid strain GSY82 is homozygous wild-type for the *HO* gene (*HO*/*HO*), the resulting spore products will be diploids, due to mating type switching. The *S. cerevisiae* diploid strain used is homozygous mutant for the *HO* gene (*ho*/*ho*), and thus the resulting spore products are haploids.

**Table 3:** Primers for distinguishing between sc (*S. cerevisiae*) and sp (*S. paradoxus*) sequences on chromosomes 6, 7, 9, and 12.

Primer	Sequence
chr06_L_sc_for	AGATGTGACTAACGTGGTGC
chr06_R_sc_for	AACCTTTATGGGGGCAATGG
chr07_L_sc_for	CCGATATAAAGTTCAGCGCC
chr07_R_sc_for	CCATCGTATTCCTGCTCTTC
chr09_L_sc_for	TTTGGACGCCTTCTCTACAG
chr09_R_sc_for	CCCTTTGGAATGTTTTCGCC
chr12_L_sc_for	CCTTTTCGTAATCAGCGTGG
chr12_R_sc_for	CCGTAAAGAAACGTCCTGTG
chr06_L_sc_rev	GAAAAGATGCGACTACTGCC
chr06_R_sc_rev	CGCTTAGTGCCAGAAATTCG
chr07_L_sc_rev	CTATCCGAGGTTGTGTACTG
chr07_R_sc_rev	GTGTTCTGTTATCACCCCAG
chr09_L_sc_rev	GGGACTTTTAGCAGTTTGCC
chr09_R_sc_rev	GTTATTTCACAGGGCCAGAC
chr12_L_sc_rev	GACAATGGCAGGAGATAACG
chr12_R_sc_rev	GGCCTAAGAATAACAGTGCG
chr07_L_sp_for	TGAAATGGGGGATGCGTTAG
chr09_L_sp_for	CTGGTGGGATTTTATGAGCG
chr09_R_sp_for	ATCGGTTGTGAATATCGGCG
chr12_L_sp_for	AAGGCACTGTAAAACACGGC
chr12_R_sp_for	GATTTCAGCCAACACTGAGC
chr07_R_sp_for	CCCATCTTACTCGCTTCTTG
chr06_R_sp_for	CCGAATCCTCAGTAGTATGC
chr06_L_sp_for	TATTCAGTAGGCAGCAGTCG
chr07_L_sp_rev	GCCGGATAGAACTAGAGATC
chr09_L_sp_rev	GAGCGATGACTAAGAAGGAC
chr09_R_sp_rev	GGAGGAGCATAGAAAAACGC
chr12_L_sp_rev	CTGAGCAGTTATCTTTCCGC
chr12_R_sp_rev	CGTACCTATTGCCTTACCTG
chr07_R_sp_rev	CTCGTTCGTGATCTATTCGG
chr06_R_sp_rev	GTTTGCTAAGAGGTGCTGAC
chr06_L_sp_rev	GCGGGCTTAAAAATGAGTGC

L: left of centromere.

R: right of centromere.

### Generation of F_1_ hybrids

*S. cerevisiae* and *S. paradoxus* strains were mated to generate either mismatch repair proficient or mismatch repair deficient F_1_ zygotes by mixing the specified strains listed in Table 2 on YPD plates for 2–3 hours.

To generate the mismatch proficient F_1_ hybrid strain, zygotes were isolated using a micromanipulator (Carl Zeiss MicroImaging, Inc., Thornwood, NY), and selecting for prototrophs. The F_1_ hybrids were confirmed by PCR for the presence of both parental genomic DNA sequences on 4 chromosomes (chromosomes 6, 7, 9, and 12).

To generate mismatch repair deficient F_1_ hybrids an *msh2::KAN* and *leu2Δ S. cerevisiae* spore and a diploid *msh2::KAN/msh2::KAN* and *ura3-1/ura3-1 S. paradoxus* were used. The *S. paradoxus msh2* mutant was sporulated for 3 days before mass mating by mixing with the *S. cerevisiae msh2* mutant on YNB plates with no supplementation. Surviving prototrophic colonies were confirmed to be F_1_ hybrids by checking for the presence of both parental chromosomes at two loci (chr6 and chr7); two independent F_1_ hybrids were kept for further use.

### Generation of F_1_ spores

F_1_ spores were generated by sporulating a diploid F_1_ hybrid in sporulation medium for 3 days with aeration. Random spore analysis [49] was performed to isolate potential viable spores of the F_1_ hybrids. Due to possible F_1_ diploid contamination in the random spore analysis, every colony was assayed for the presence of *S. cerevisiae* or *S. paradoxus* chromosomes (2 loci each on chromosomes 6, 7, 9, and 12 for a total of 8 loci). If a clone contained both parental copies of all 4 chromosomes, then it was assumed to be a surviving F_1_ diploid (rather than being a strain that is aneuploid for all 4 chromosomes), and was not used for further analysis.

### Dual-species array design

The contig sequences of *S. paradoxus* and the genomic sequences of *S. cerevisiae* were downloaded from the *Saccharomyces* Genome Database [50]. Each contig and chromosome was divided into 2 kb segments. ArrayOligoSelector [51] was used to find 60 mer probe sequences for each of the 2 kb segments from each organism, using the combined sequences of *S. paradoxus* and *S. cerevisiae* genomes as a mask, to eliminate cross hybridization potential, either within or between species. The parameters used were: 38% GC, 60 mers, up to 3 oligonucleotides per segment. The oligo sequences produced by ArrayOligoSelector were blasted against the mask file using blastn with e-score cutoff of 1×10^−10^. The oligonucleotides having more than one match were eliminated. For each segment that had more than one oligonucleotide, the oligonucleotide with the lowest Gibb's free energy of binding was chosen, unless the GC content was outside of the 30–50% range, then the oligonucleotide with the more optimal GC content was chosen. The oligonucleotides that had more than one hit as determined by the ArrayOligoSelector program were also eliminated. The gap distance between adjacent probes was minimized by re-running ArrayOligoSelector on the largest gap regions to find additional oligonucleotides. In addition, we designed oligonucleotide probes for control sequences used by van de Peppel *et al* [52] using this same approach.

### Array CGH and analysis

Genomic DNA was isolated and purified using the YeaStar yeast genomic DNA kit (Zymo Research, Orange, CA) and then quantified using the Qubit fluorometer (Invitrogen, Carlsbad, CA). The genomic DNA was fragmented with *Hae*III (New England Biolabs, Ipswitch, MA) at 37°C for 1 hour, and the products were purified using Microcon-30 columns (Millipore, Billerica, MA). For all arrays, a mixture of equal molar amounts of *S. cerevisiae* and *S. paradoxus* genomic DNA was used as reference. The fragmented genomic DNA of an F_1_ spore and the reference genomic DNA mix were differentially labeled using the Ulysis labeling kit with Alexa fluors 546 and 647 (Invitrogen, Carlsbad, CA) following manufacturer's instructions and hybridized to the custom dual-species Agilent arrays (Agilent Technologies, Santa Clara, CA). The arrays were washed and scanned following manufacturer's instructions.

For the pooling experiments, after sporulation and germination, roughly 1000 viable F_1_ spores were picked with sterile toothpicks and combined for genomic DNA extraction and subsequent array CGH analysis. Independent sporulations and pooling experiments were performed for both mismatch repair proficient and deficient F_1_ hybrids.

The software Feature Extraction v 9.1.5 (Agilent Technology, Santa Clara, CA) was used to extract and normalize the microarray data using a LOWESS based normalization. The normalized arrayCGH results are presented as log base 10 ratios of hybrid genomic DNA over the reference. The results are visualized using the software Caryoscope [27]. *S. paradoxus* contigs were mapped to the *S. cerevisiae* genome by blasting the contigs against the *S. cerevisiae* chromosomes; the contig order was then used to create an input file for Caryoscope wherein the chromosomes were collinear between the species.

### Data analysis

#### Identification of recombination locations

Each of the probes was aligned along the chromosomes for each of the two species. To determine whether the segment of a chromosome in a particular species, for which the probe was designed to detect, was present or absent in a given F_1_ spore, the log ratios were converted to binary values, such that if a probe had a log_10_ ratio of less than an −0.05 (visually, this threshold value worked best for our dataset), then it was assigned a value of 0, and if had a log_10_ ratio of greater than −0.05, then it was assigned a value of 1. Using these data, each chromosome was then analyzed for the presence of recombination events by dividing each chromosome into regions containing a minimum of 10 probes, with >70% of these probes having the same value (as defined above). If more than one region was identified within a chromosome, then one or more recombination events was inferred to have occurred within the chromosome, and the borders between the regions were designated as the recombination locations. A chromosome of a particular parental origin was assumed to be completely absent if 80% or more of the probes from that chromosome had log_10_ ratios of less than an arbitrarily chosen threshold of −0.05. Each recombination location identified by this algorithm was validated via visual inspection of the data using Caryoscope.

#### Segmentation of each chromosome

After the recombination locations were identified for each chromosome for each F_1_ spore, the data for all the F_1_ spores to be analyzed were combined. For each chromosome, all the recombination events that were observed in any of the F_1_ spores were recorded. Each chromosome for each F_1_ spore was segmented based on these recorded recombination locations (irrespective of whether a given F_1_ spore had a recombination event occur at these locations). After segmentation, each segment for each F_1_ spore was scored for their presence (1), absence (0), or aneuploidy (2) in the particular strain. Each parental species' chromosome was analyzed separately. Aneuploidy was scored based on the presence of the segment from both species. No cases of whole chromosome aneuploidy were observed, where a viable spore had two copies of a chromosome derived from one of the parental species.

#### Linkage analysis

Pairwise linkage analysis was performed for all possible pairs of segments across all 16 chromosomes. The segments for each species were analyzed separately. For each pair of segments A and B within a particular F_1_ spore, the four categories analyzed were (for *S. cerevisiae*): both segments present from the *S. cerevisiae* (1/1), segment A is present from *S. cerevisiae* while segment B is present from *S. paradoxus* (1/0), vice versa (0/1), and both segments A and B are from *S. paradoxus* (0/0). For each pair of segments, the total number of F_1_ spores that were assigned to each category was recorded. Chi squared statistics were calculated for each possible pairs of segments. The uncorrected p-values for each pair were calculated using 3 degrees of freedom.

The false discovery rate (FDR) for each p-value was estimated by permuting the dataset, such that the number of strains with a particular segment from one or the other species, or aneuploid was preserved, but the strains that had inherited each particular segment were randomized. Chi square statistics for the four pairwise comparisons as stated above were calculated for the randomized dataset and the number of pairs of segments with a specific p-value was calculated. A total of 1000 randomized datasets were generated. The FDR for a specific p-value, *x*, was calculated as the number of pairs of segments with p-values less than or equal to *x* among the 1000 randomized datasets divided by the average number of pairs of segments with p-values less than or equal to *x* in the real dataset.

#### Local sequence identity calculations

Each pair of homeologous chromosomes was aligned using LAGAN [53]. The alignment results were used to calculate the local sequence identities using sliding windows of 100 bp in size.

### Data availability

All data have been deposited in the GEO database with accession number GSE19683.

## Supporting Information

Figure S1 Comparisons between sequence identity between *S. cerevisiae* and *S. paradoxus* and GC content and frequency of recombination. Gray lines: sequence identity between *S. cerevisiae* and *S. paradoxus*. Light blue: percent local GC content for *S. cerevisiae*. Green: Percent local GC content for *S. paradoxus*. Brown: frequency of meiotic recombination in *S. cerevisiae*. Open purple triangles: frequency of observed recombination in wild-type F_1_ spores. Open pink squares: frequency of observed recombination in mismatch repair mutant F_1_ spores.
(1.53 MB PDF)

Figure S2 Chi-square statistics versus distance between intrachromosomal segments.
(0.63 MB PDF)

Table S1 Comparisons between percent identity, recombination frequencies, and local GC content between *S. cerevisiae* and *S. paradoxus*.
(0.02 MB XLS)

Table S2 Pairwise linkage analysis results for *S. cerevisiae* portion of the genome.
(8.09 MB ZIP)

Table S3 Pairwise linkage analysis results for *S. paradoxus* portion of the genome.
(8.12 MB ZIP)

Table S4 Statistically significant pairs of interchromosomal regions in the *S. cerevisiae* genome using chi-square test and an FDR of 0.01.
(0.04 MB XLS)

Table S5 Statistically significant pairs of interchromosomal regions in the *S. paradoxus* genome using chi-square test and an FDR of 0.01.
(0.05 MB XLS)

Table S6 Significant pairs of loci in *S. cerevisiae* containing pairs of genes with known or predicted interactions.
(0.03 MB XLS)
