# Supplementary figures and images for: A Genome-Wide Analysis Reveals No Nuclear Dobzhansky-Muller Pairs of Determinants of Speciation between S. cerevisiae and S. paradoxus, but Suggests More Complex Incompatibilities
Source: PLoS Genet. 2010 Jul 29;6(7):e1001038. doi: 10.1371/journal.pgen.1001038 (PMC2912382; doi:10.1371/journal.pgen.1001038)

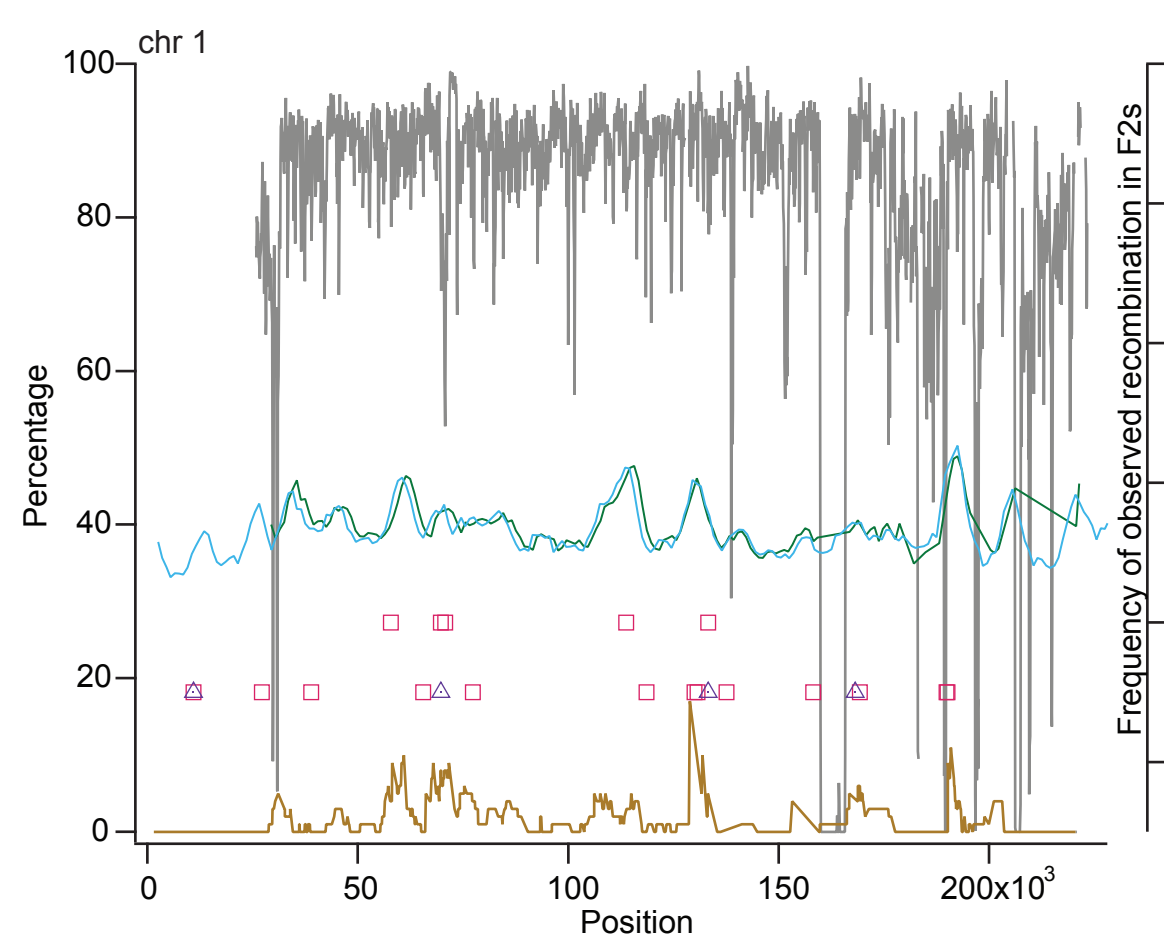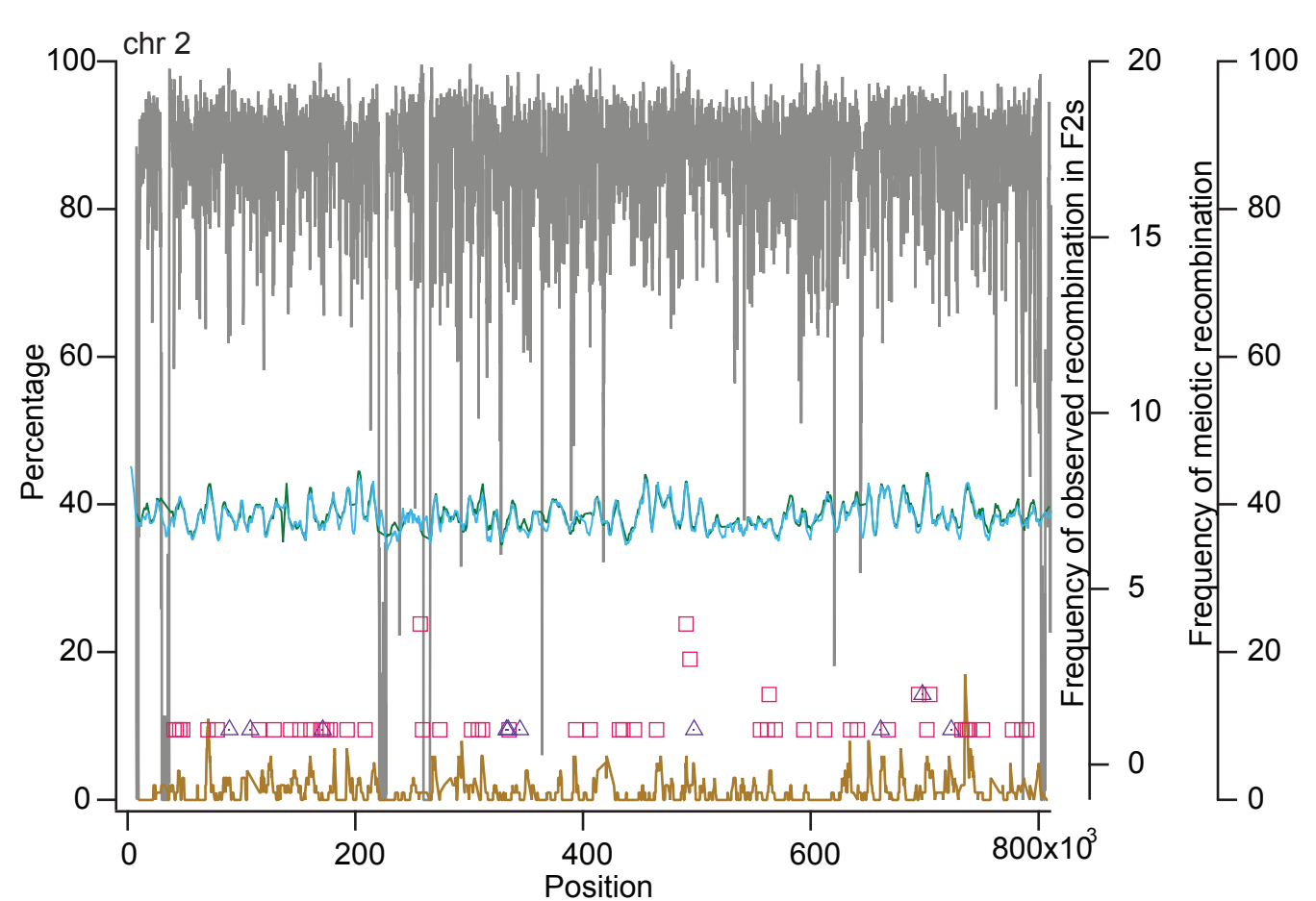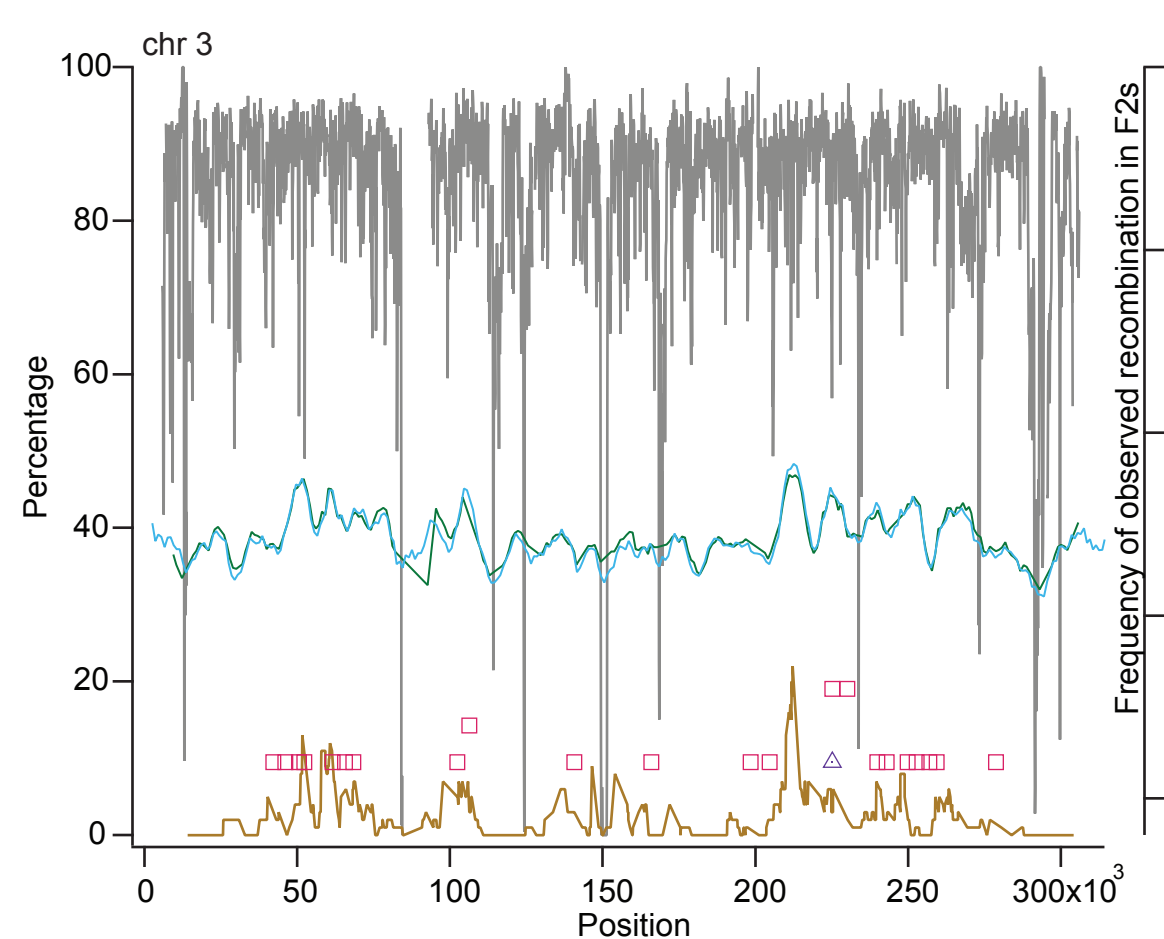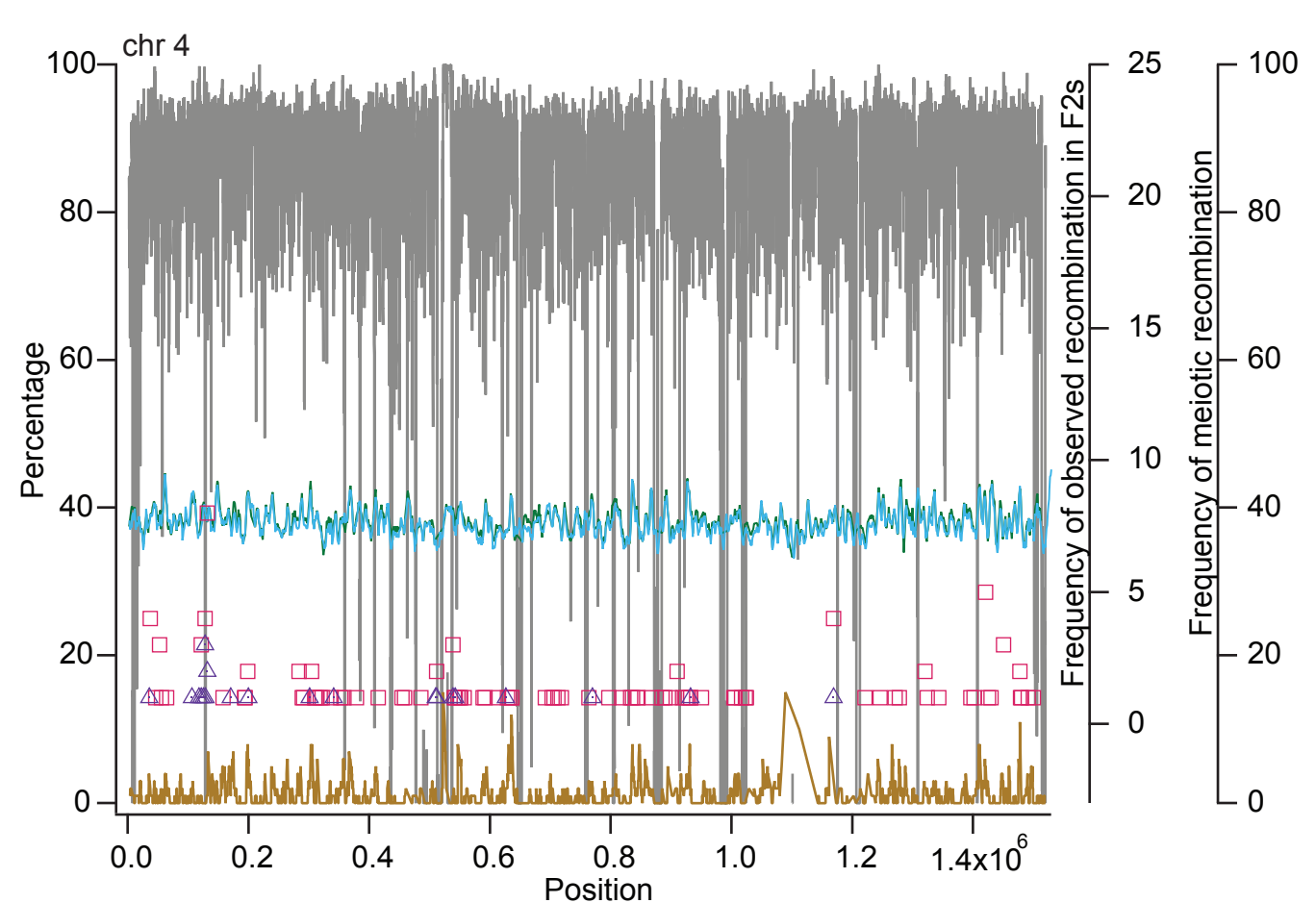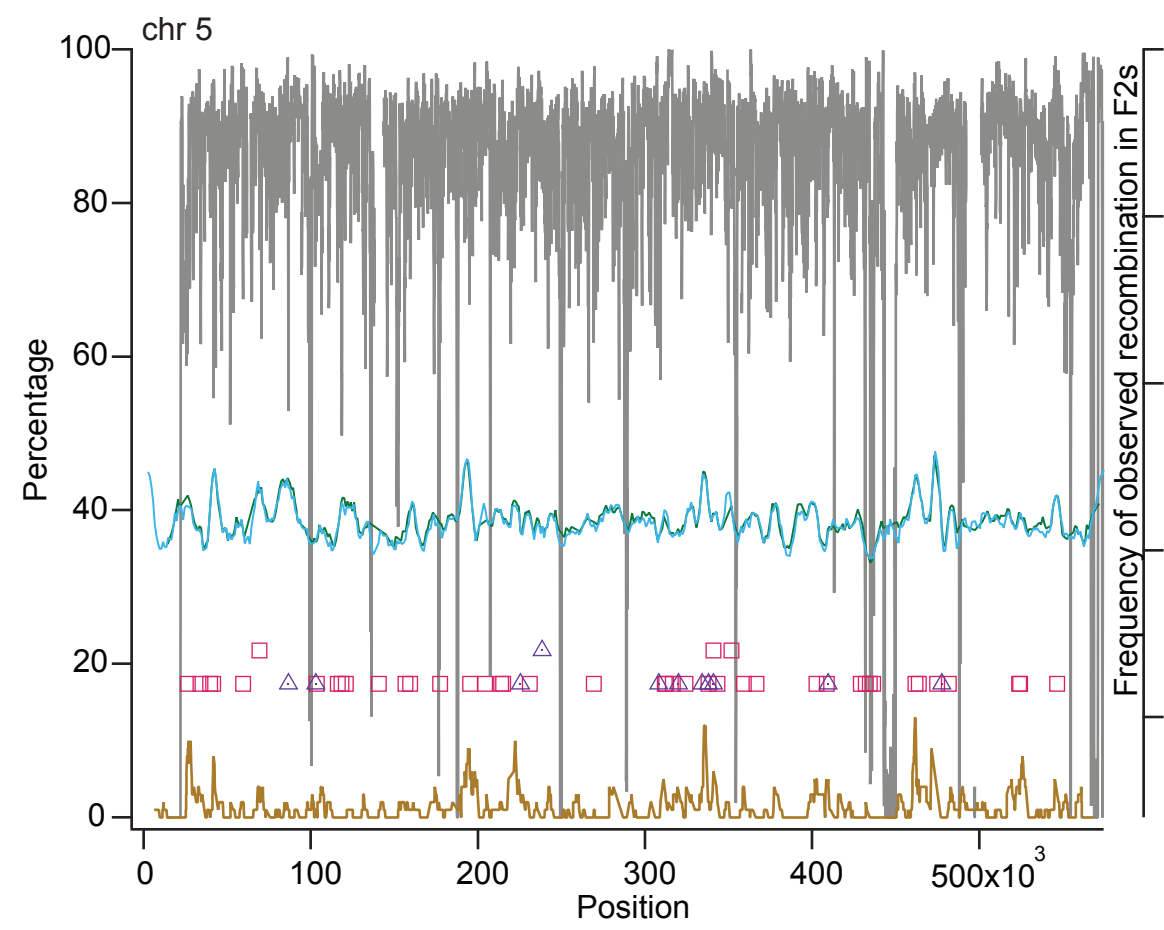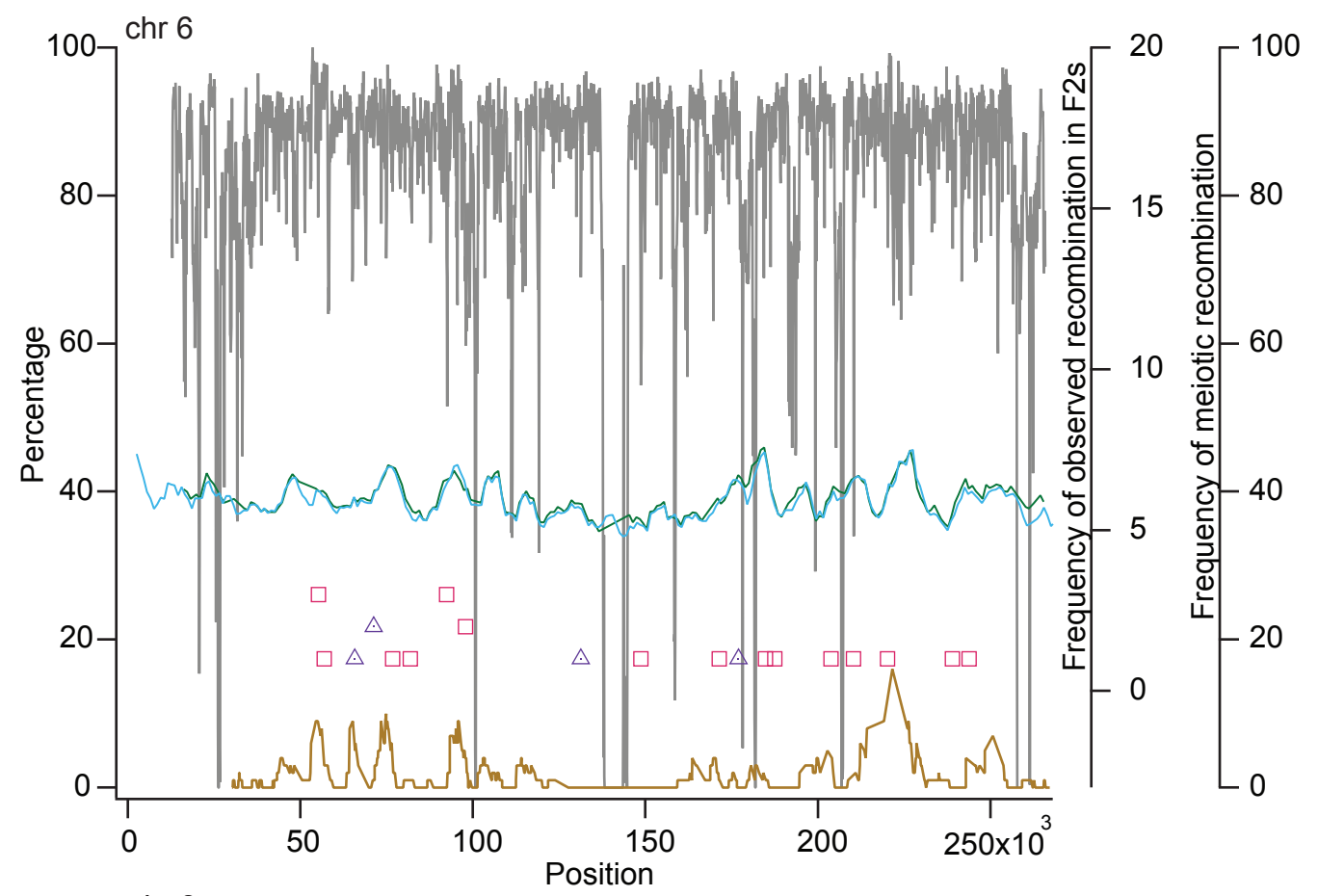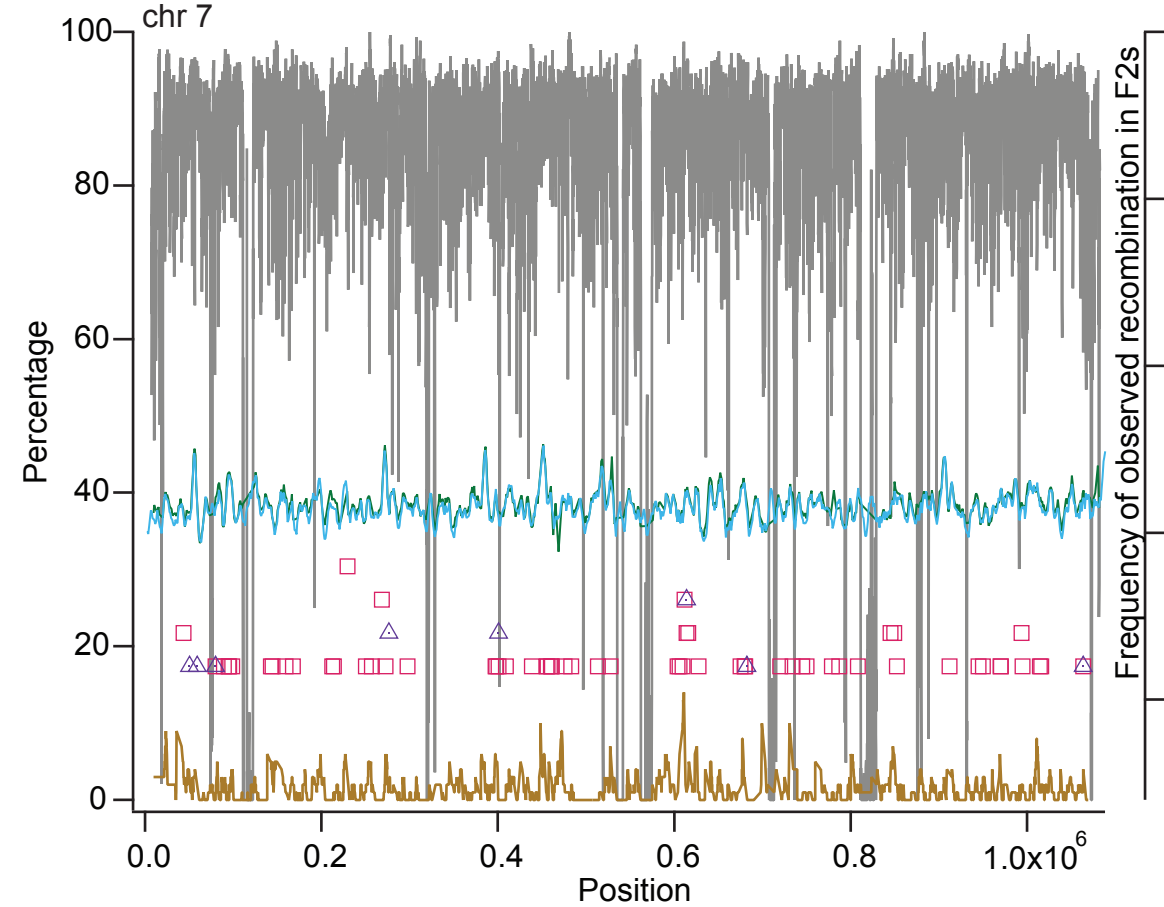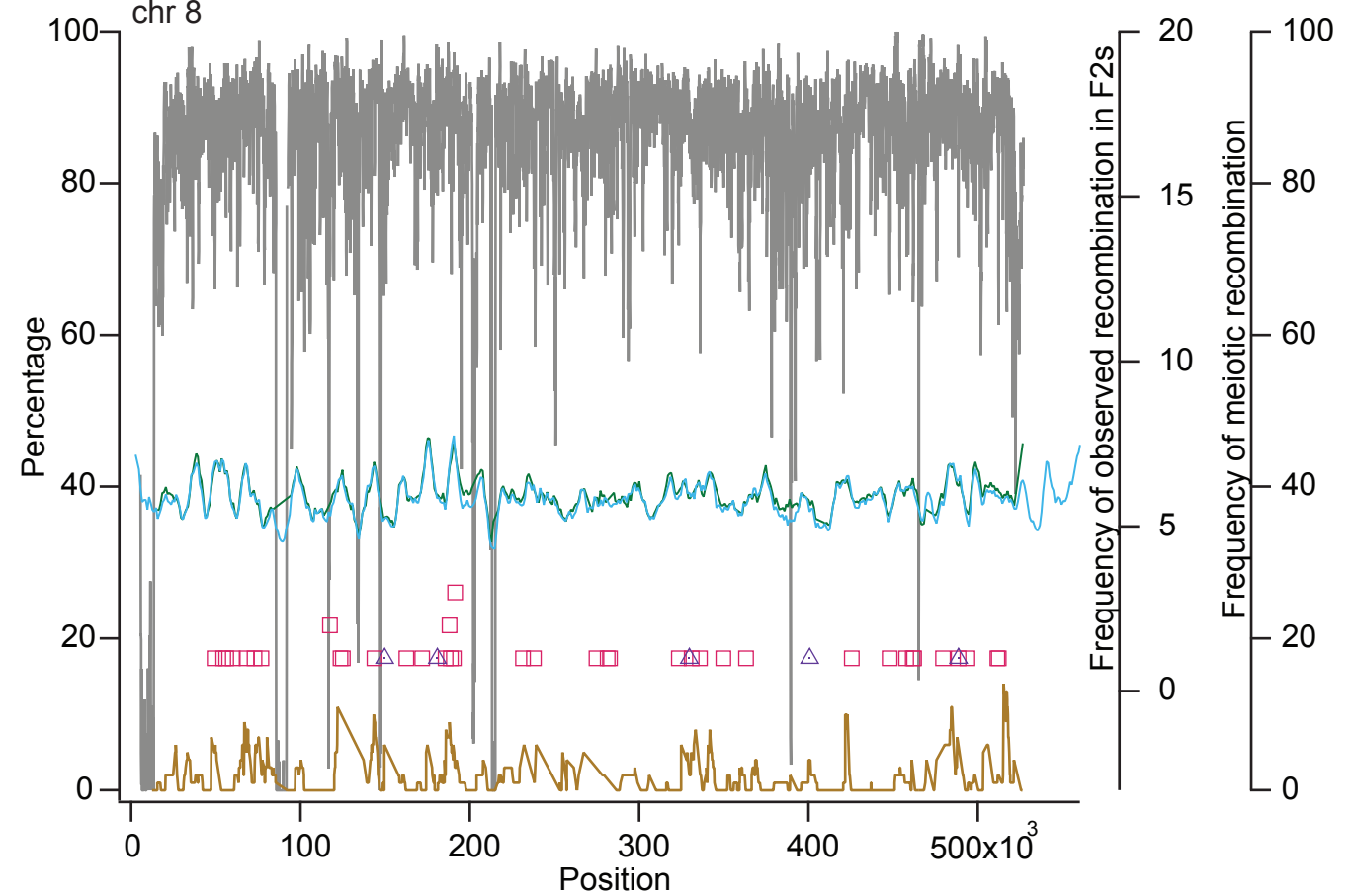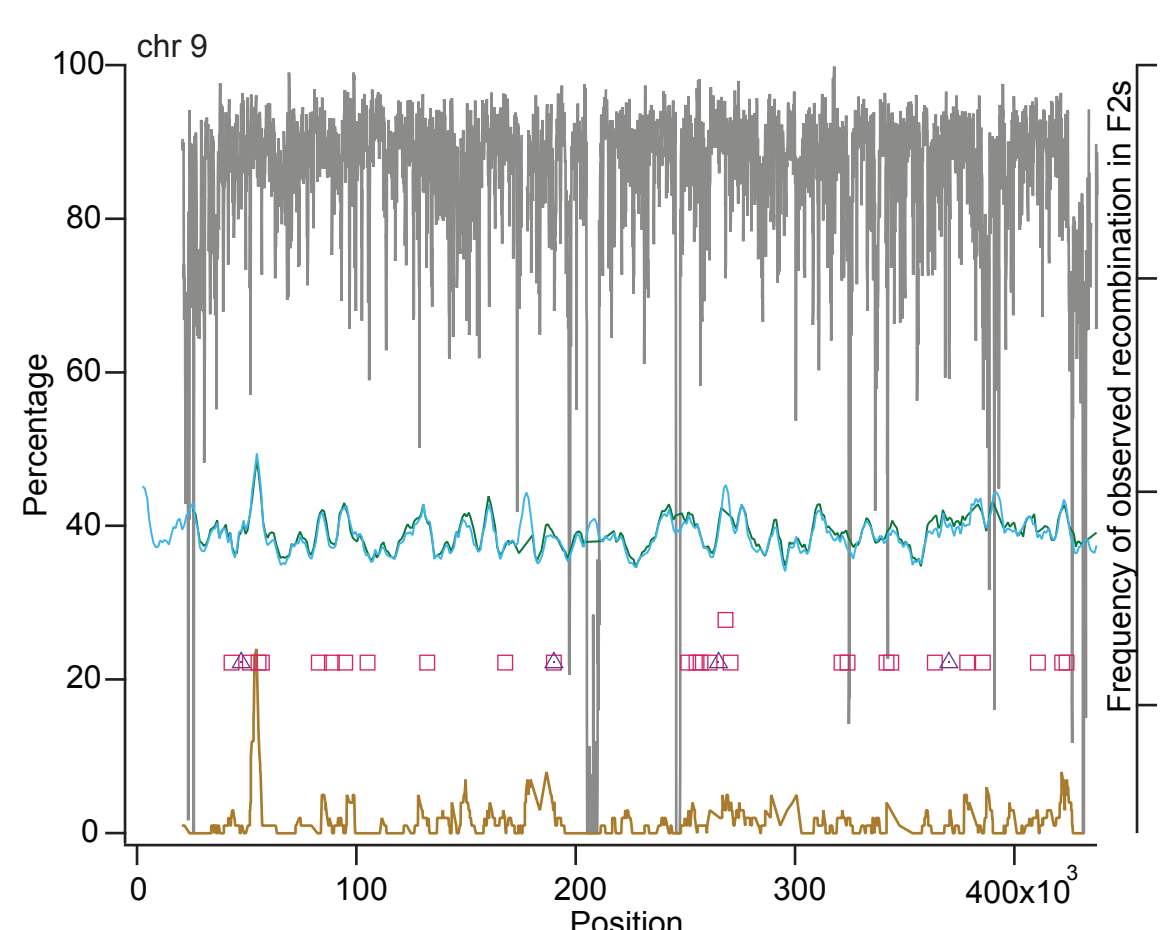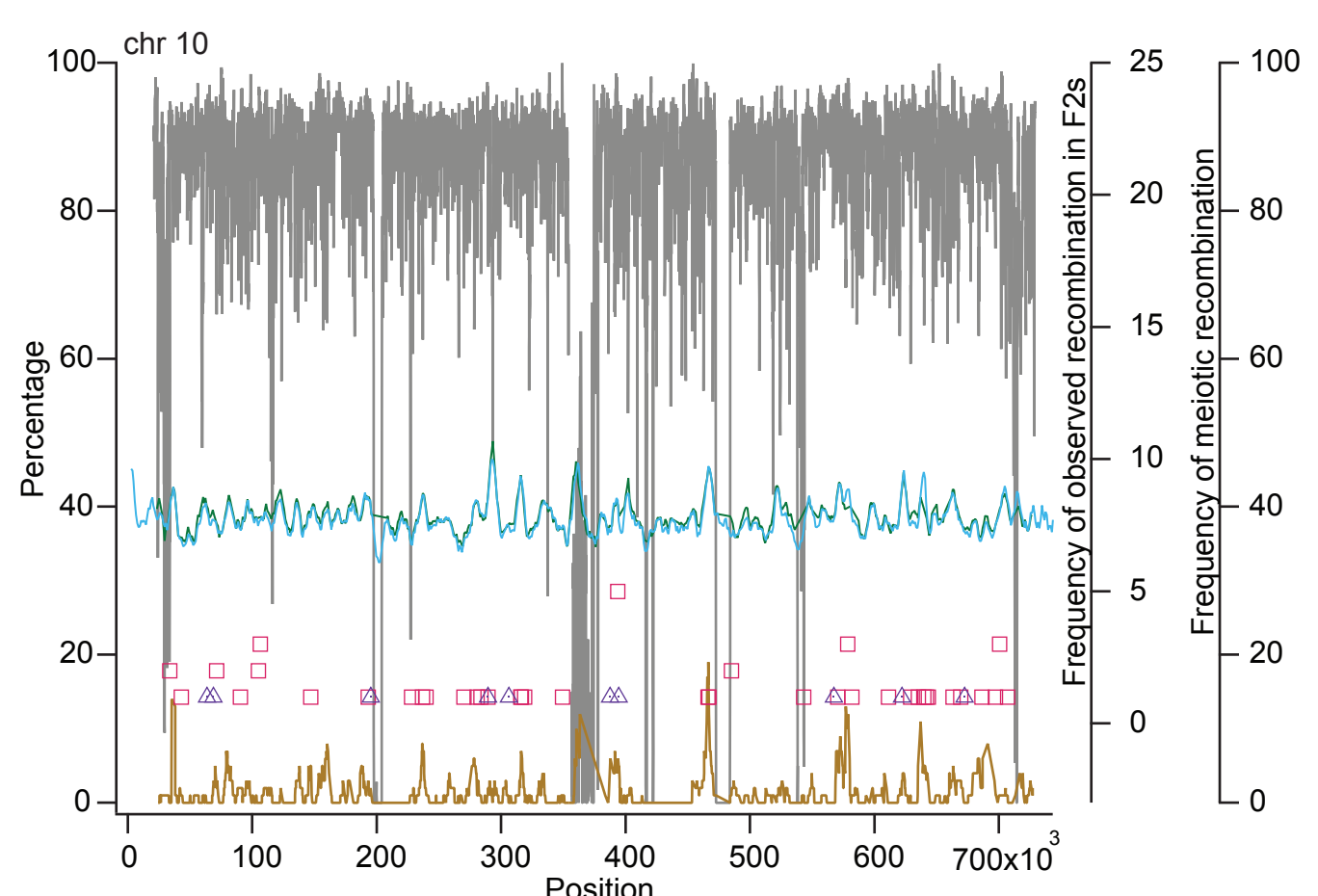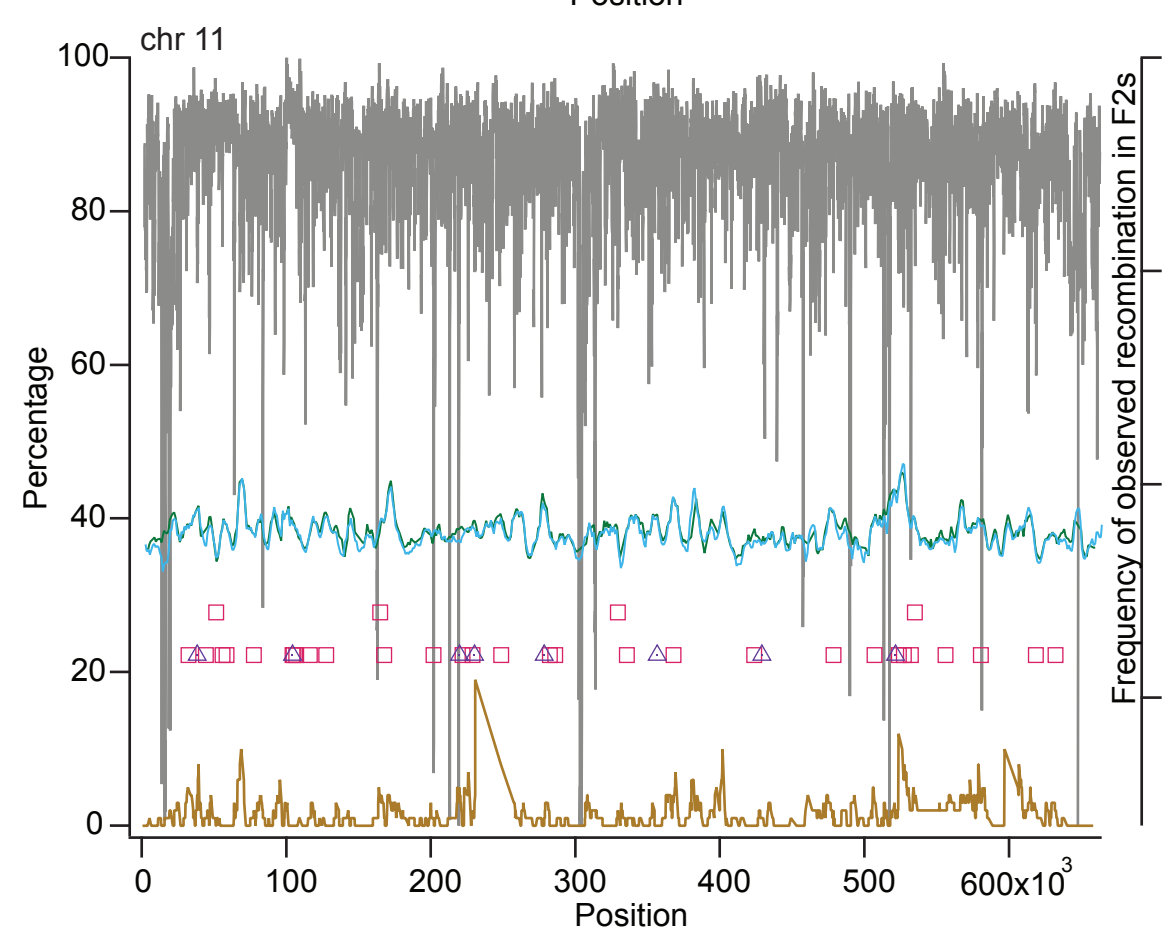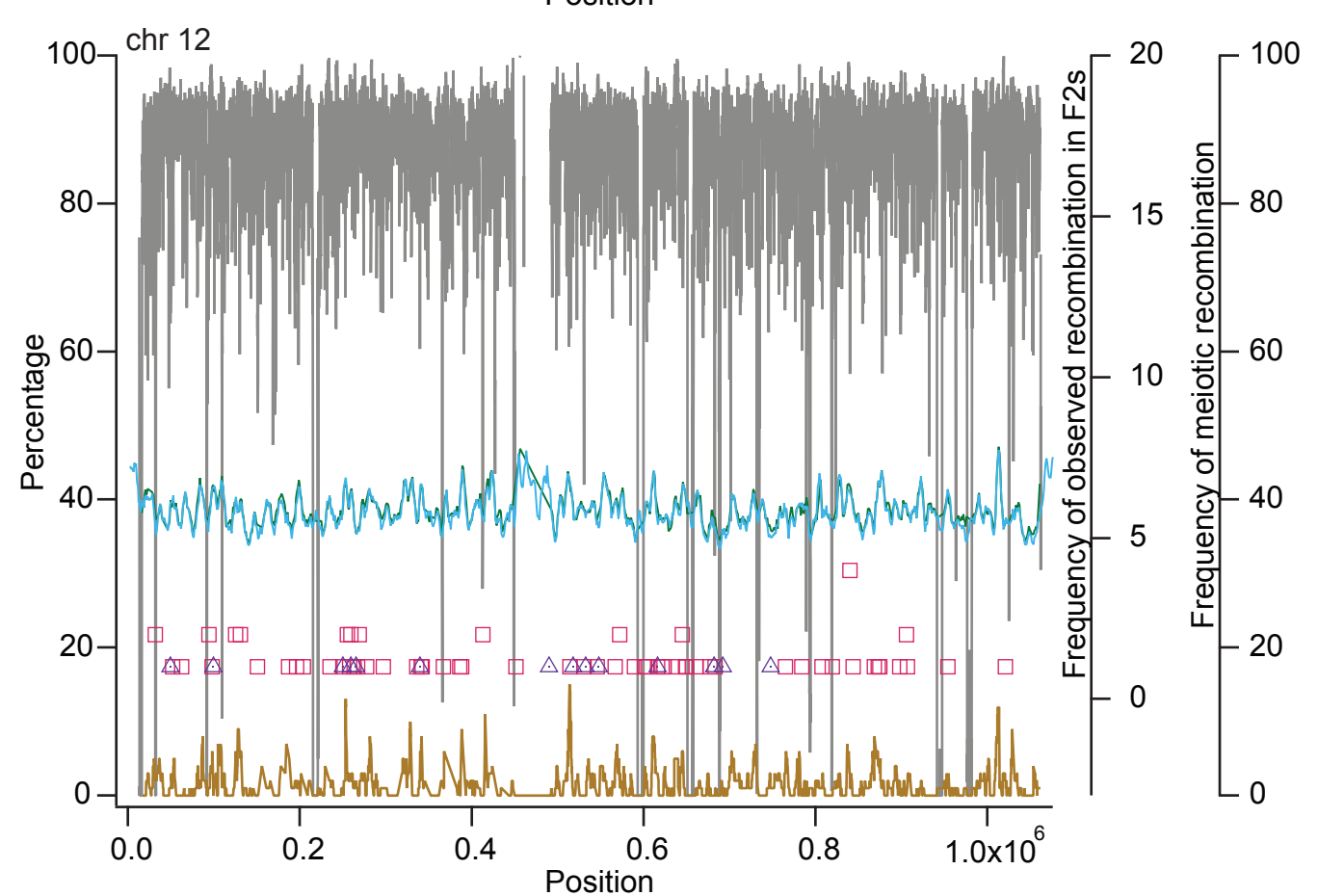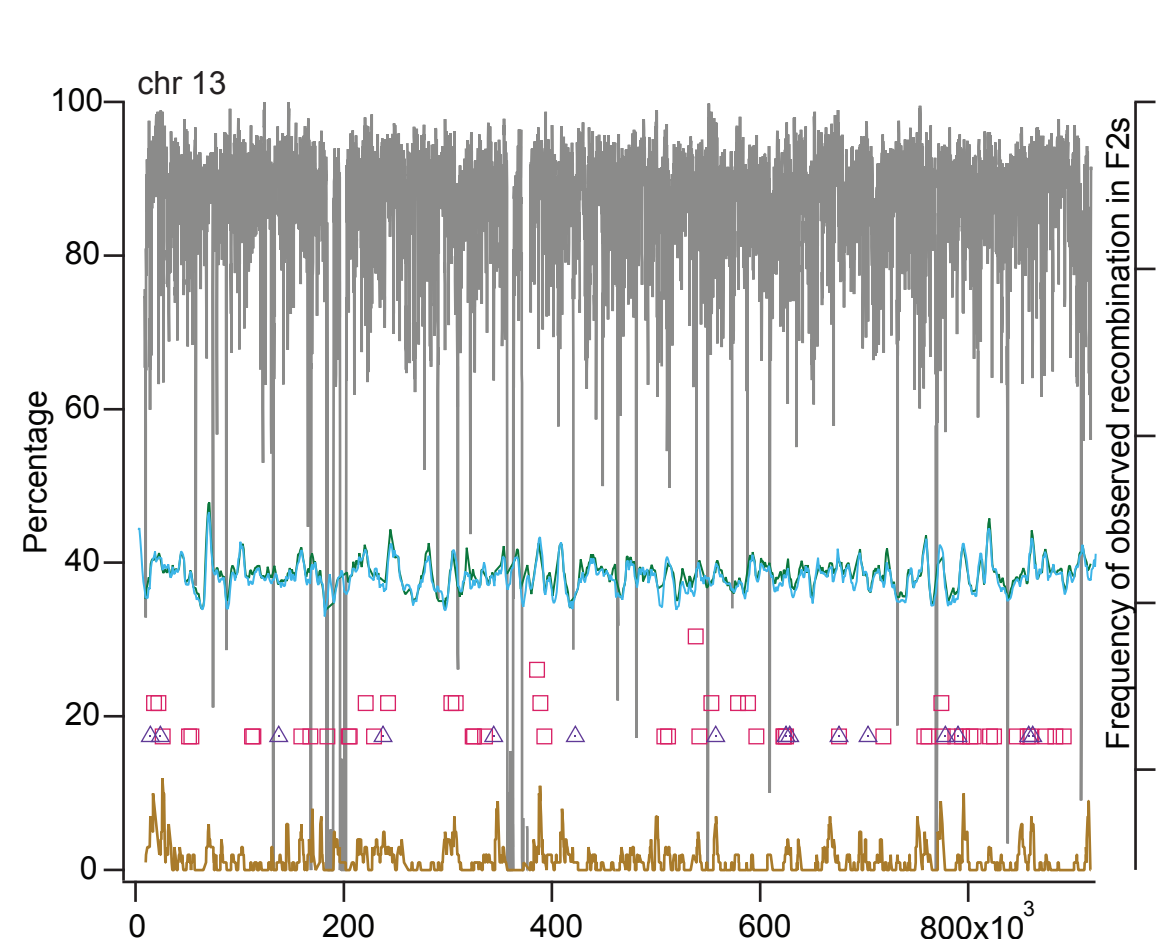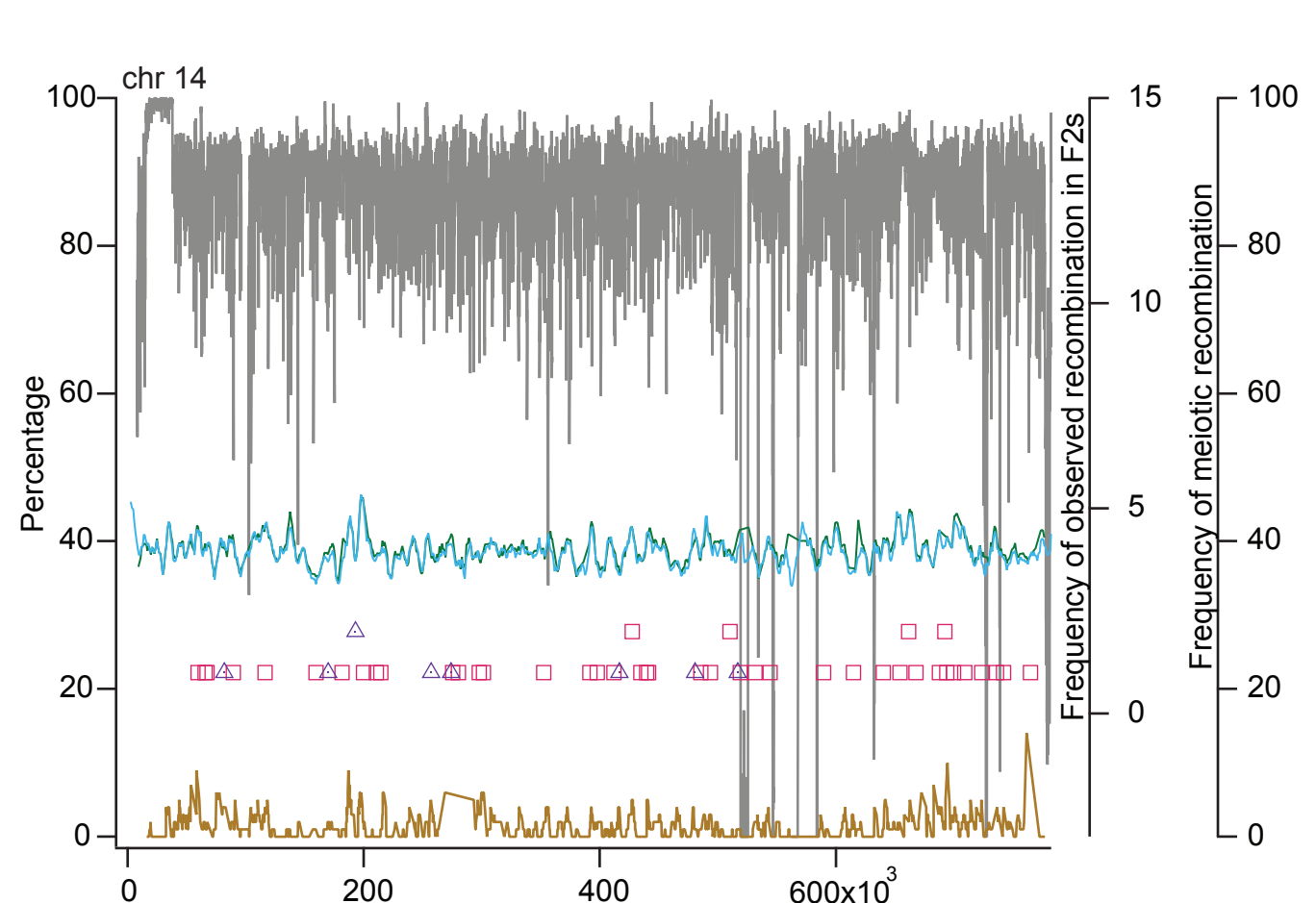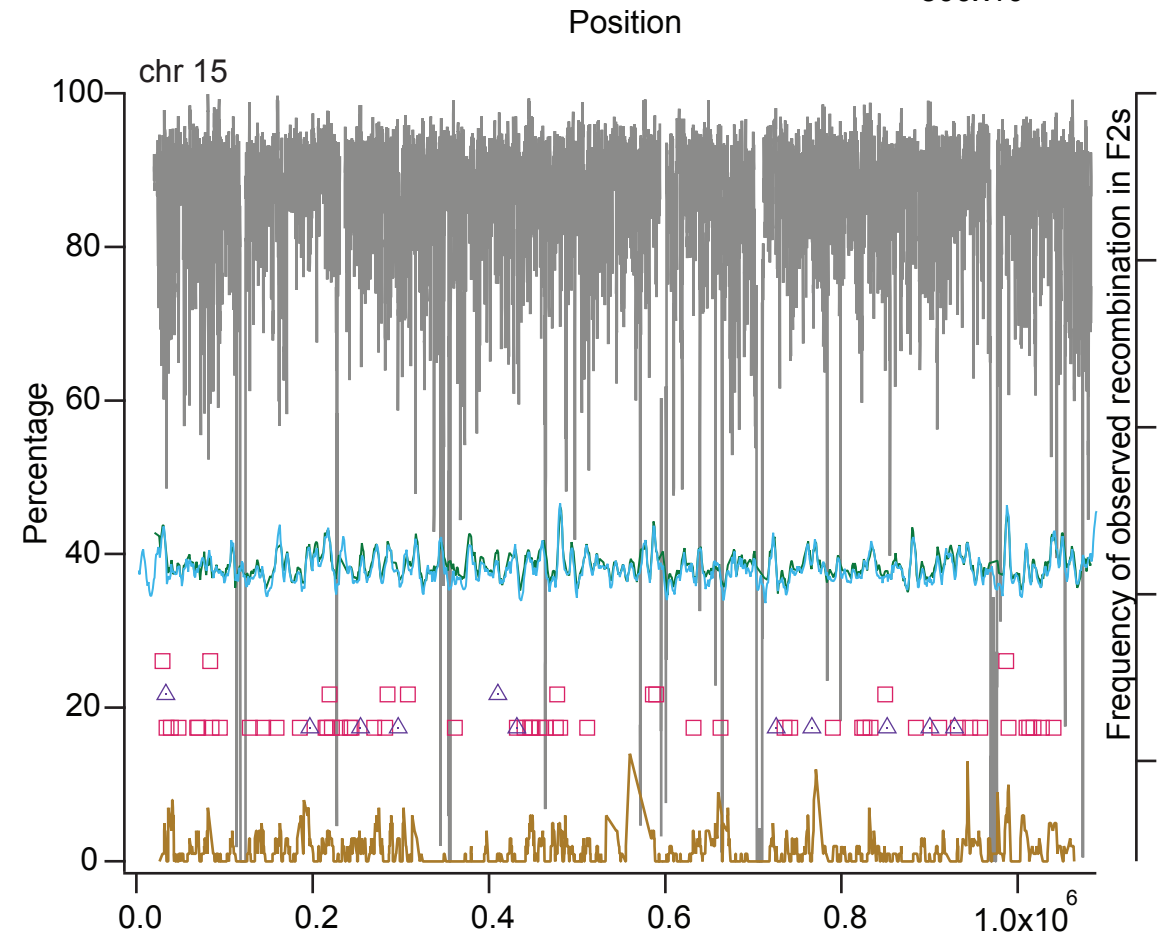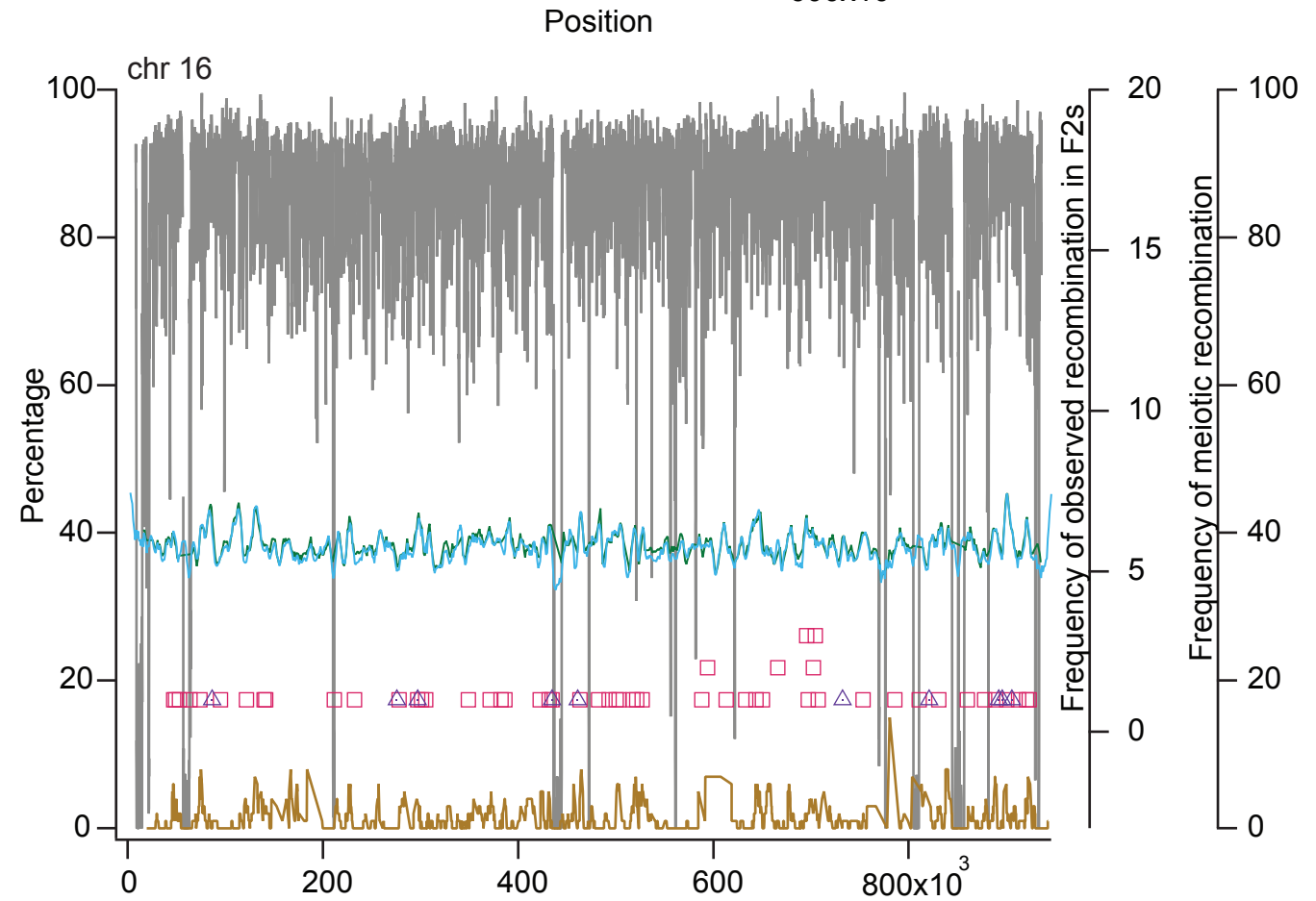

Supplement: Figure S1 — Comparisons between sequence identity between S. cerevisiae and S. paradoxus and GC content and frequency of recombination. Gray lines: sequence identity between S. cerevisiae and S. paradoxus. Light blue: percent local GC content for S. cerevisiae. Green: Percent local GC content for S. paradoxus. Brown: frequency of meiotic recombination in S. cerevisiae. Open purple triangles: frequency of observed recombination in wild-type F1 spores. Open pink squares: frequency of observed recombination in mismatch repair mutant F1 spores. (1.53 MB PDF) [file pgen.1001038.s001.pdf]

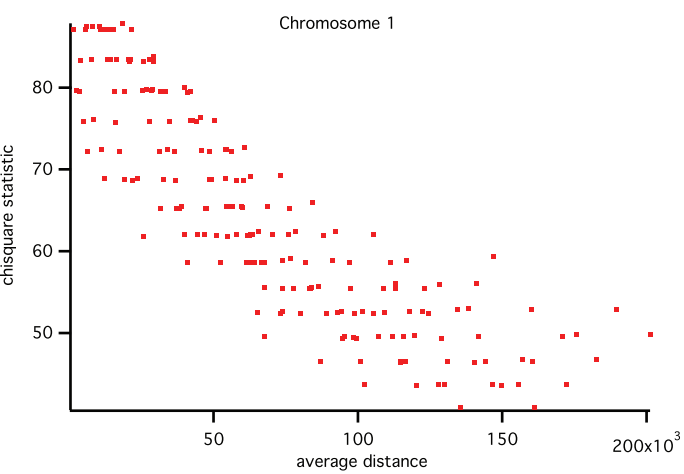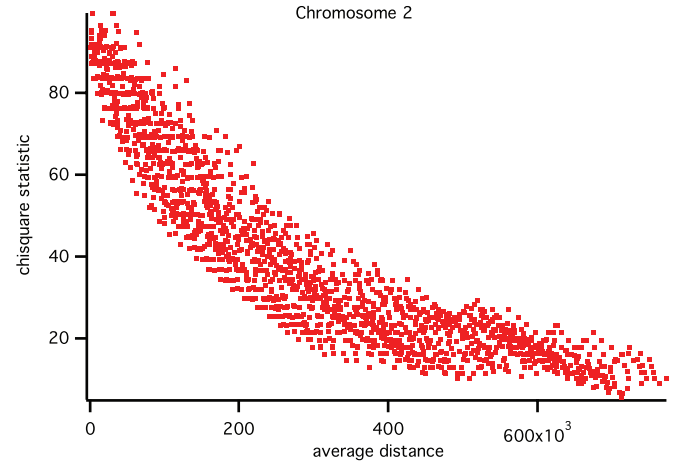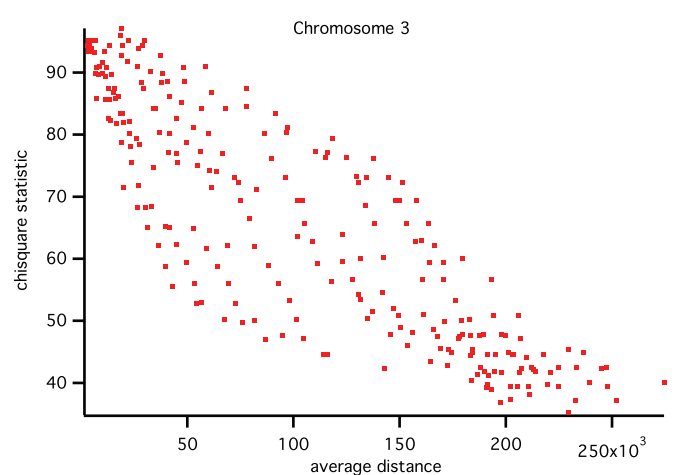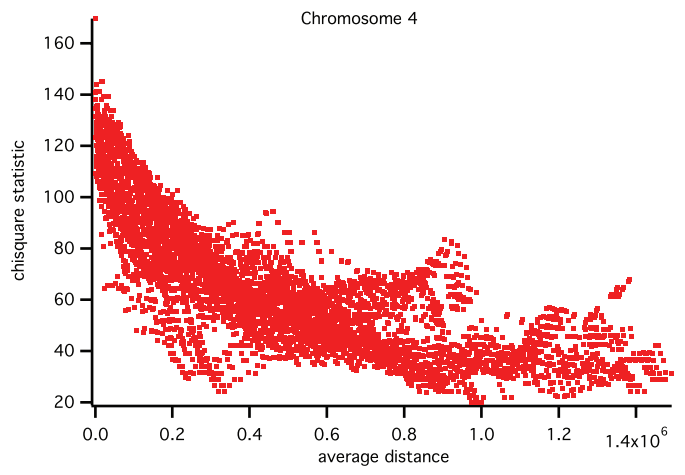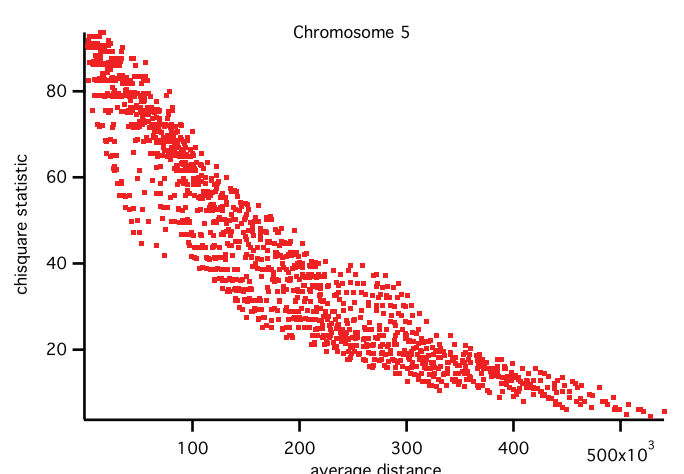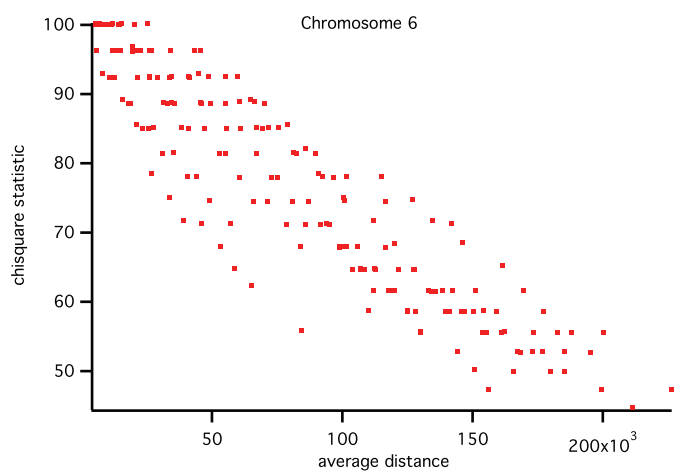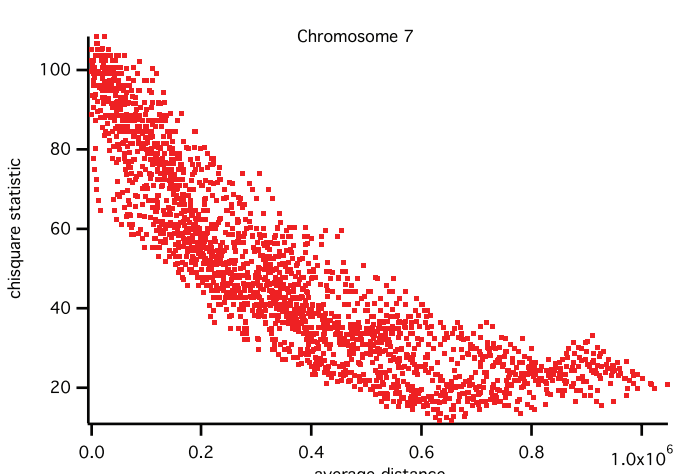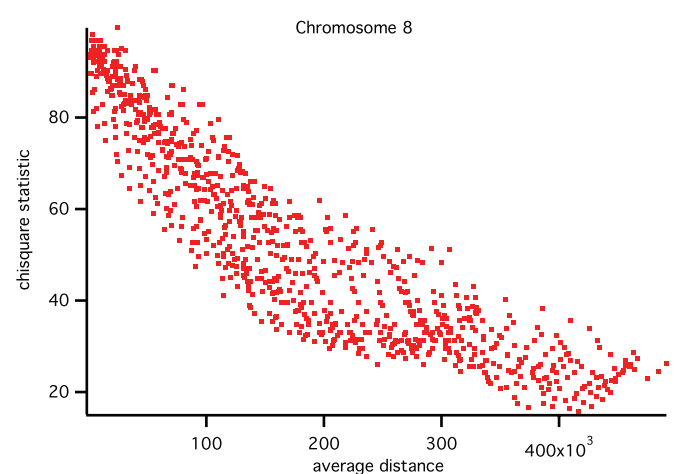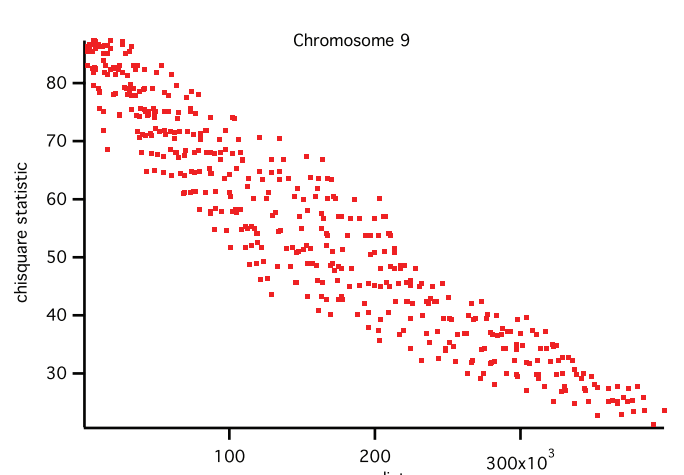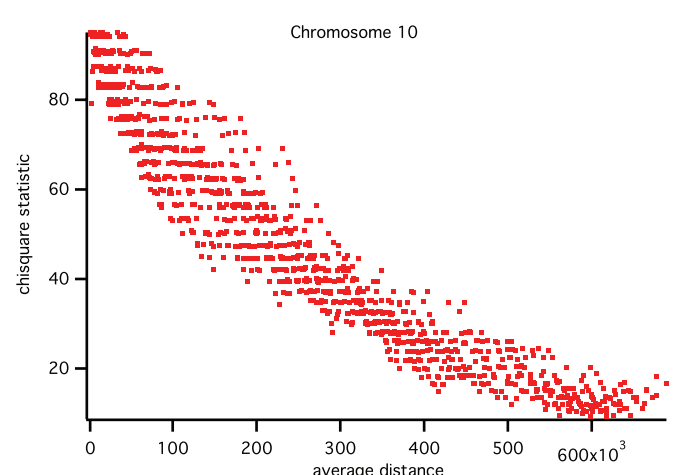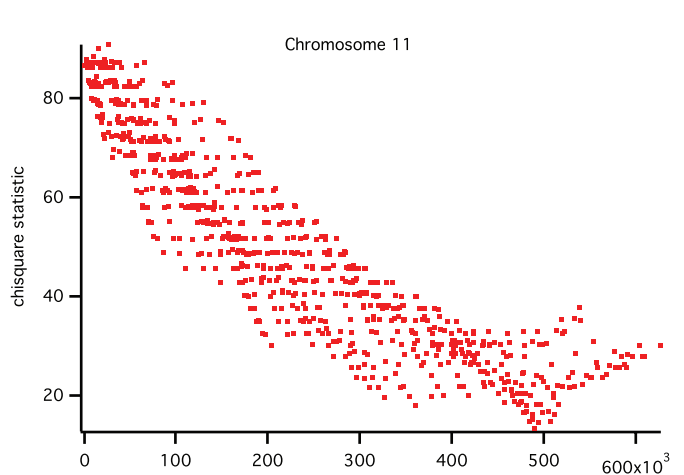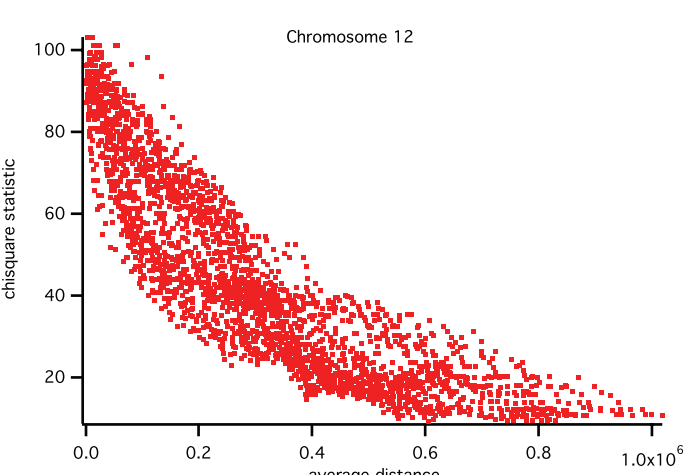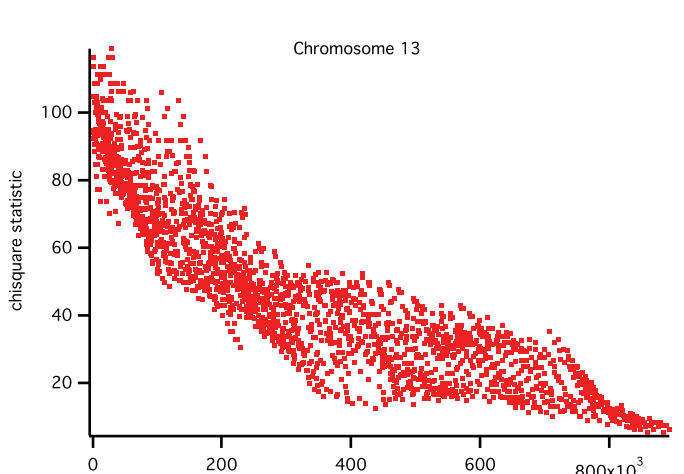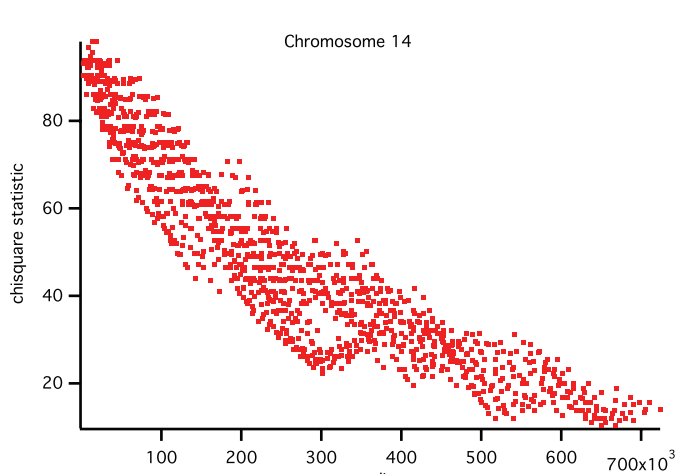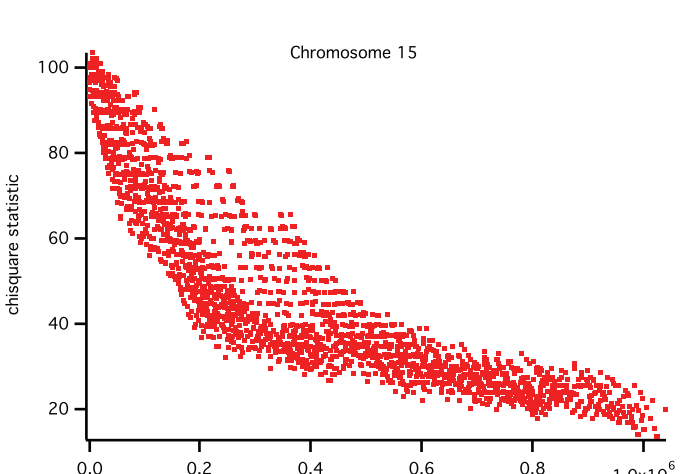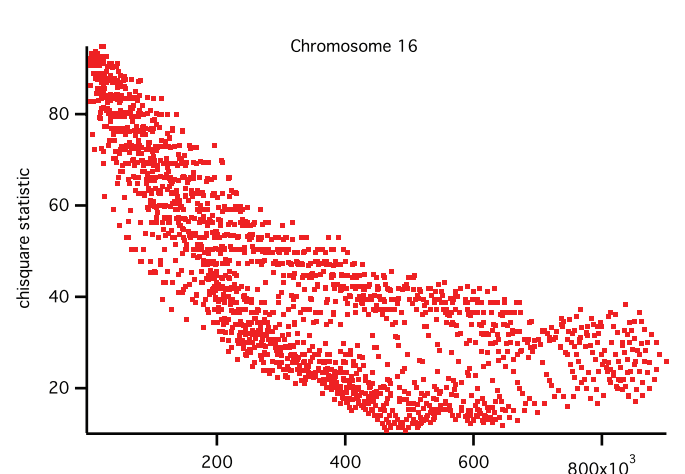

Supplement: Figure S2 — Chi-square statistics versus distance between intrachromosomal segments. (0.63 MB PDF) [file pgen.1001038.s002.pdf]
